# Rejuvenation of white adipose tissue in a longitudinal heterochronic transplantation model

**DOI:** 10.64898/2025.12.28.696721

**Published:** 2025-12-29

**Authors:** Alibek Moldakozhayev, Alexander Tyshkovskiy, Pasquale Nigro, Cecília G. de Magalhães, Kejun Ying, Alec W. Eames, Jesse R. Poganik, Albina Tskhay, Bohan Zhang, Stanislav Tikhonov, Praju Vikas Anekal, Nicholas P. Carbone, Michael F. Hirshman, Laurie J. Goodyear, Jeremy M. Van Raamsdonk, Vadim N. Gladyshev

**Affiliations:** 1 Division of Genetics, Department of Medicine, Brigham and Women’s Hospital, Harvard Medical School, Boston, MA, USA; 2 Broad Institute of MIT and Harvard, Cambridge, MA, USA; 3 Department of Neurology and Neurosurgery, McGill University, Montreal, QC, Canada; 4 Metabolic Disorders and Complications, and Brain Repair and Integrative Neuroscience Programs, Research Institute of the McGill University Health Centre, Montreal, QC, Canada; 5 Section on Integrative Physiology and Metabolism, Joslin Diabetes Center, Boston, MA, USA; 6 Stanford University School of Medicine, Stanford, CA, USA; 7 Institute for Protein Design, University of Washington, Seattle, WA, USA; 8 Department of Family Medicine, McGill University, Montreal, QC, Canada; 9 CHU Sainte-Justine Research Centre, Montreal, QC, Canada; 10 Faculty of Bioengineering and Bioinformatics, Lomonosov Moscow State University, Moscow, Russia; 11 Belozersky Institute of Physico-Chemical Biology, Moscow State University, Moscow, Russia; 12 MicRoN Core, Harvard Medical School, Boston, MA, USA; 13 Harvard Medical School, Boston, MA, USA; 14 Division of Experimental Medicine, Department of Medicine, McGill University, Montreal, QC, Canada

## Abstract

Exposure to a younger system can induce organismal rejuvenation, yet whether all tissues can be rejuvenated and by what mechanisms remains understudied. We performed heterochronic and isochronic transplantation of subcutaneous white adipose tissue (WAT) between young and old mice and longitudinally tracked changes in biological age. Transplantation accelerated tissue aging, and the molecular age of grafts shifted toward that of the host. Most importantly, old WAT was rejuvenated in a young body. Epigenetic and transcriptomic clocks revealed a reduction of predicted age, accompanied by coordinated activation of canonical and previously unrecognized thermogenic pathways. Molecular rejuvenation was further supported by architectural changes toward a youthful state, including reduced lipid droplet size and decreased cellular heterogeneity. Mitochondrial abundance and morphology remained unchanged, while collagen deposition increased. These results demonstrate that WAT biological age is partially reversible and identify molecular and cellular features underlying its rejuvenation

## Introduction

Aging is characterized by the progressive accumulation of deleterious molecular transformations that ultimately drive disease onset and organismal death [1–4]. Age-related diseases, such as cancer, cardiovascular disorders, and neurological conditions, pose significant health and economic challenges and are among the leading causes of disability and mortality in our society [5]. Given that aging itself is a key cause of developing age-related diseases [6], targeting the aging process offers a promising way to delay the onset of diseases [7, 8], consequently extending healthspan and life expectancy of humans.

While slowing aging may delay the onset of age-related diseases, rejuvenation offers the potential for more radical anti-aging approaches by not only treating existing conditions but also preventing their future emergence. Heterochronic parabiosis (HPB), in which the circulatory systems of a young and an old animal are surgically joined, has long been considered a powerful experimental model of rejuvenation. Studies have demonstrated that exposure of an old animal to a youthful systemic environment can improve molecular and functional features of aging in multiple tissues, such as muscle, liver, heart, brain, and bone, highlighting the potential of a young system to ameliorate age-associated decline [9–15]. The most recent research employing epigenetic and transcriptomic profiling to assess biological age has shown that HPB can enhance physiological functions, reduce epigenetic and transcriptomic ages, restore youthful gene expression profiles, and ultimately extend the lifespan of older mice, even after the animals were separated [16, 17].

Despite compelling evidence from heterochronic parabiosis that systemic factors can partially reverse organismal aging, it remains unclear whether all tissues share comparable rejuvenation potential. Most studies have focused on whole-organism phenotypes or “canonical” tissues such as blood and liver, leaving tissue-specific rejuvenation largely unexplored. A direct way to address this gap is to transplant individual old organs or tissues into young hosts and assess the extent to which their biological age can be reset. However, transplantation of vital organs, particularly when pre-sampling is required, risks compromising tissue integrity and function. To overcome these challenges, we leveraged subcutaneous white adipose tissue (WAT) - a non-vital, easily accessible depot that can be sampled prior to transplantation without substantial risk. This feature makes WAT uniquely suited for longitudinal assessment of biological age and provides a tractable model to study the intrinsic rejuvenation capacity of aged tissues *in vivo*.

WAT is a highly dynamic endocrine organ essential for energy homeostasis and systemic metabolic regulation [18, 19]. In addition to serving as a primary site of lipid storage, WAT secretes a broad array of adipokines that influence organismal metabolism and coordinate the function of other tissues. With age, WAT undergoes marked dysfunction characterized by impaired progenitor activity, increased infiltration of senescent and pro-inflammatory cells, and a redistribution of fat from subcutaneous to visceral depots [18]. These deleterious changes are accompanied by a decline in the thermogenic capacity of the tissue, driven by the gradual loss of beige and brown adipocytes - cell populations associated with better metabolic health. The result is insulin resistance, chronic low-grade inflammation, and ectopic lipid accumulation. Given that adipose tissue is among the earliest sites to manifest age-associated decline, it may act as a critical driver of systemic metabolic deterioration, thereby accelerating organismal aging [18, 19].

Global demographic and metabolic trends underscore the medical relevance of these changes. Obesity has become one of the most pressing health challenges worldwide and is now among the leading contributors to morbidity and mortality [20, 21]. Its intersection with population aging is particularly alarming: the number of individuals over 60 is expected to nearly double by 2050, and around 40% of adults over 60 are expected to be classified as obese [22, 23]. Because WAT is both a central metabolic organ and one of the earliest tissues to exhibit age-associated decline, aging and obesity converge to place older obese individuals at exceptional risk for metabolic dysfunction - most notably type 2 diabetes (T2D), which is strongly linked to impaired WAT biology [18, 24]. Unraveling the mechanisms that drive WAT aging and establishing whether these changes can be reversed, therefore, represents a critical step toward mitigating metabolic deterioration in an increasingly aged and obese global population.

A central unresolved question is whether aged WAT can regain youthful molecular and functional features when placed in a young systemic environment, and which biological programs mediate such transformation. To address this, we established a longitudinal murine adipose tissue transplantation model in which old WAT was engrafted into young hosts, and reciprocally, young WAT into old hosts, to directly test the impact of systemic environment on tissue aging trajectories. We quantified biological age using both epigenetic and transcriptomic clocks and validated molecular insights with histological assessments of tissue architecture. This experimental framework allowed rigorous evaluation of whether a young systemic environment can rejuvenate intrinsically aged tissue and enabled dissection of the molecular pathways and structural outcomes underlying this process, extending the paradigm of young-blood–mediated rejuvenation to a central metabolic and endocrine organ.

## Results

### Transplantation of white adipose tissue

**Figure 1.**
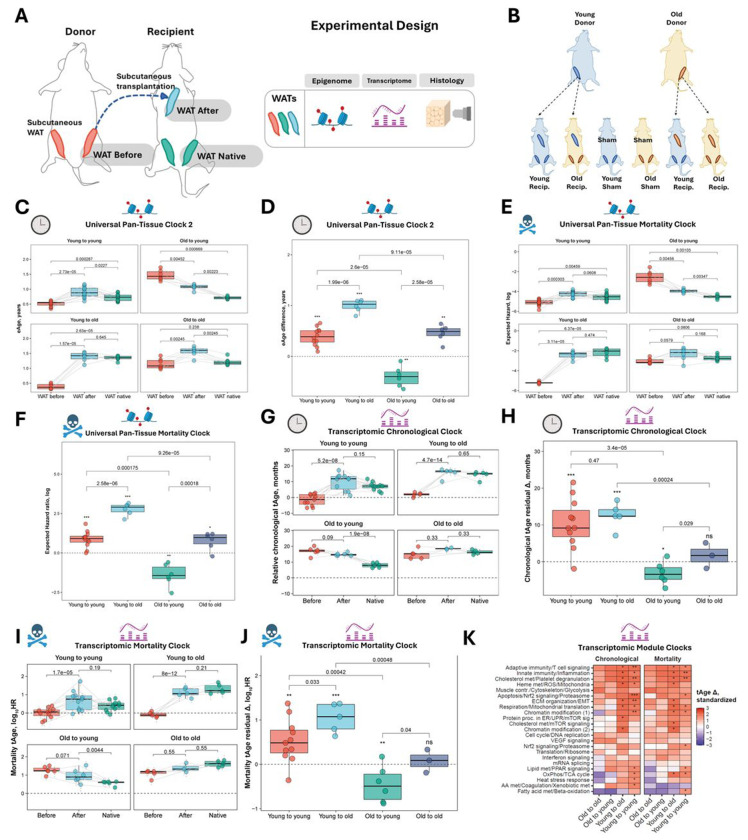
Epigenetic and transcriptomic aging clocks reveal rejuvenation of old adipose tissue following heterochronic transplantation. **(A)** Overview of the study design. Subcutaneous inguinal white adipose tissue (WAT) was harvested prior to transplantation (WAT Before), following transplantation (WAT After), and from untouched regions of the same animals (WAT Native) for epigenetic, transcriptomic, and histological profiling. **(B)** Schematic of the experimental setup. Heterochronic (old-to-young, young-to-old) and isochronic (young-to-young, old-to-old) transplantations were performed, with tissues collected before and after grafting. **(C)** Epigenetic age (eAge) of WAT samples before and after transplantation across all experimental groups according to the Universal Clock 2 predictions. Mouse ID was included in the statistical model as a covariate to ensure paired comparison of eAge dynamics. **(D)** Change in epigenetic age (eAge) of WAT samples before and after transplantation across all experimental groups according to Universal Clock 2. Asterisks reflect statistical significance (BH-adjusted p-values) of eAge changes during transplantation within individual groups, whereas the significance of pairwise comparisons in eAge dynamics between groups is denoted in text. **(E)** Expected hazard ratios (in log scale) of WAT samples before and after transplantation across all experimental groups according to the epigenetic pan-tissue Mortality Clock. Mouse ID was included in the statistical model as a covariate to ensure paired comparison of expected hazard ratio dynamics. **(F)** Change in expected hazard ratios (in log scale) of WAT samples before and after transplantation across all experimental groups according to the Epigenetic Mortality Clock. Asterisks reflect statistical significance (BH-adjusted p-values) of expected hazard ratio changes during transplantation within individual groups, whereas the significance of pairwise comparisons in expected hazard ratio dynamics between groups is denoted in text. **(G)** Transcriptomic age (tAge) of WAT samples before and after transplantation across all experimental groups according to the Bayesian Ridge (BR) rodent multi-tissue Chronological Clock. Mouse ID was included in the statistical model as a covariate to ensure paired comparison of tAge dynamics. Statistical significance was assessed with mixed-effects models. Data are tAges ± s.d. **(H)** Age-adjusted change in transcriptomic age (tAge) of WAT samples before and after transplantation across all experimental groups, according to the rodent multi-tissue Transcriptomic Chronological Clock. Statistical significance was assessed with mixed-effects models. Asterisks reflect statistical significance (BH-adjusted p-values) of tAge changes during transplantation within individual groups, whereas the significance of pairwise comparisons in tAge dynamics between groups is denoted in text. **(I)** Expected mortality (in log scale) of WAT samples before and after transplantation across all experimental groups according to the rodent multi-tissue Transcriptomic Mortality Clock. Mouse ID was included in the statistical model as a covariate to ensure paired comparison of tAge dynamics. Statistical significance was assessed with mixed-effects models. Data are tAges ± s.d. **(J)** Age-adjusted change in expected mortality (in log scale) of WAT samples before and after transplantation across all experimental groups, calculated by the rodent multi-tissue Transcriptomic Mortality Clock. Statistical significance was assessed with mixed-effects models. Asterisks reflect statistical significance (BH-adjusted p-values) of expected mortality changes during transplantation within individual groups, whereas the significance of pairwise comparisons in expected mortality dynamics between groups is denoted in text. **(K)** Standardized change of tAge in WAT samples following transplantation across all experimental conditions, according to module-specific rodent multi-tissue transcriptomic chronological and mortality clocks. Red color indicates increased tAge, while blue color indicates decreased tAge after transplantation relative to their respective pre-transplantation baselines. Asterisks reflect statistical significance (BH-adjusted p-values) of changes. Boxplots: center line indicates median; box limits, interquartile range; whiskers, ±1.5× IQR. Unless specified otherwise, statistical differences between groups are assessed with ANOVA and adjusted for multiple comparisons with the Benjamini-Hochberg approach. *** p.adjusted< 0.001, ** p.adjusted < 0.01, * p.adjusted < 0.05.

To explore the potential for adipose tissue rejuvenation and to dissect aging trajectory differences, we transplanted subcutaneous inguinal WAT into the subcutaneous compartment in the interscapular region of recipient mice. We collected WAT samples from (1) donor animals prior to transplantation, (2) the same WAT from the graft site 10 weeks post-transplantation, and (3) the untouched inguinal WAT of recipients, enabling within-animal longitudinal comparisons of aging dynamics. Each WAT type was subjected to comprehensive molecular and structural profiling, specifically epigenetic, transcriptomic, and histological analyses (Figure 1A).

To rigorously evaluate the potential to rejuvenate WAT, we had multiple control groups enabling isolation of the specific reciprocal effects of the host and graft age on long-term aging and rejuvenation of the tissues. Specifically, in addition to the primary experimental group, young recipients of old fat, we included young recipients of young fat, old recipients of young fat, and old recipients of old fat, allowing us to determine the influence of host age on the graft age during heterochronic transplantation. We also included sham surgery controls to delineate the effects of surgery on WAT transplantation. The full experimental design is summarized in Figure 1B.

### Epigenetic and transcriptomic aging clocks reveal rejuvenation of old adipose tissue following heterochronic transplantation

To quantify biological age changes in transplanted adipose tissue, we applied two advanced multi-tissue pan-mammalian epigenetic age estimators, Universal Clocks 2 and 3 [25], to collected WAT samples (Figure 1C, Supplementary Figure 1A). In isochronic transplantations (young-to-young and old-to-old), we observed a significantly increased predicted age in post-transplant grafts (WAT After) relative to both their baseline states (WAT Before) and the untouched adipose tissue from recipient animals (WAT Native), across both clocks. In heterochronic young-to-old transplants, young adipose tissue placed into old hosts acquired a biological age indistinguishable from that of the host’s own Native WAT, indicating that the aged systemic environment rapidly imposes an old epigenetic state on young grafts. In contrast, old adipose tissue implanted into young recipients underwent substantial rejuvenation, reflected by a marked and significant reduction in predicted age.

The longitudinal design of our study, which included pre-transplant clock measurements, allowed us to directly assess the magnitude and direction of biological age changes for individual grafts using Universal Clock 2 (Figure 1D), and Universal Clock 3 (Supplementary Figure 1B). Subtracting “WAT Before” from “WAT After” predicted values revealed that isochronic grafts significantly aged to a similar extent regardless of donor or host age following the transplantation. Young tissues implanted into old bodies exhibited greater age acceleration than either isochronic group, underscoring the stronger influence of the old systemic environment on young tissue. In contrast, old adipose tissue transplanted into young hosts was the only group to demonstrate a significant reversal of aging trajectory, with changes significantly different from those seen in old-to-old and young-to-young groups, indicating the capacity of the young environment to induce rejuvenation in old tissue.

To assess whether this rejuvenation extended to epigenetic features associated with all-cause mortality, we applied the pan-mammalian Mortality Clock - a multi-species multi-tissue epigenetic predictor trained to predict animal’s expected mortality (Figure 1E). The Mortality Clock largely recapitulated the Universal Clock results, with minor distinctions: isochronic grafts exhibited modestly higher expected hazard values than native recipient WAT, although these differences did not reach statistical significance. Young grafts transplanted into old hosts were again epigenetically indistinguishable from the recipients’ native tissue. Most importantly, old WAT placed into young hosts showed a marked reduction in expected hazard values. Longitudinal analysis of within-sample changes (Figure 1F) fully mirrored the patterns observed with the Universal Clocks, reinforcing that a youthful systemic environment can reverse both chronological- and mortality-linked epigenetic features in aged adipose tissue.

Because epigenetic age predictions may be influenced by global shifts in methylation, whether due to biological factors or technical variability such as coverage of probe hybridization, we compared average methylation levels across all groups. No significant differences were observed between any comparisons (Supplementary Figure 1C), suggesting that the observed epigenetic age dynamics were not driven by global methylation shifts or technical errors. We also evaluated whether transplantation affected epigenetic noise, as measured by Shannon entropy, which has been associated with aging and molecular disorder [26]. While we observed a general trend toward increased entropy in all post-transplant adipose tissue samples, these differences were not statistically significant (Supplementary Figure 1D). Old adipose tissue transplanted into old recipients showed a marginally significant increase in entropy suggesting a possible mild increase in stochastic epigenetic noise following the transplantation.

To assess whether the rejuvenation observed at the epigenetic level extended to gene expression, we applied rodent multi-tissue transcriptomic clocks of chronological age and expected mortality [17] to a subset of the same adipose tissue samples. In the case of the Transcriptomic Chronological Clock (Figure 1G), isochronic young-to-young WAT After showed a statistically significant increase of the predicted transcriptomic age (tAge) of grafted tissues relative to WAT Before, with modest but non-significant elevations compared to Native WAT of recipients. Similarly, in old-to-old transplants, predicted transcriptomic ages of post-transplant grafts were modestly higher than both baseline and native tissue samples, but these differences did not reach statistical significance. Young adipose tissues placed into old recipients fully assimilated tAges of hosts, paralleling the findings observed with epigenetic clocks and further underscoring the dominant role of the systemic environment. Old adipose tissues transplanted into young recipients exhibited a decrease in tAges compared to baseline. While the overall reduction of transcriptomic age reached only marginal statistical significance, the direction of change aligned with the rejuvenation effect. The comparatively weaker statistical signal for the rejuvenation observed at the level of transcriptomic clocks may reflect technical factors such as batch effects introduced by separate sequencing runs for some of the samples.

To further examine tAge dynamics, we analyzed the magnitude and direction of biological age changes for individual grafts, paralleling the approach used for epigenetic clocks, by subtracting pre-transplant (WAT Before) from post-transplant (WAT After) values. Comparison of tAge changes adjusted for chronological age (i.e., age deviations) (Figure 1H) revealed that the young adipose tissue transplanted into old and young recipients exhibited similar predicted age deviation dynamics, according to the chronological transcriptomic clock. In contrast, old adipose tissue transplanted into young recipients exhibited a significant deceleration of the calculated transcriptomic age dynamics. The direction of tAge deviation changes was significantly different from both old-to-old and young-to-young controls, reinforcing the notion that exposure to a youthful systemic environment can counteract features of transcriptomic aging in old tissues.

Application of Transcriptomic Mortality Clocks to WAT Before, WAT After, and Native WAT samples yielded results nearly identical to those obtained with the chronological transcriptomic clock (Figure 1I), suggesting that the observed transcriptomic changes reflect biologically meaningful changes associated with health and mortality risk. A further analysis of tAge dynamics values adjusted for chronological age (Figure 1J) revealed that young adipose tissue transplanted into old recipients underwent a significantly greater aging rate deviation than either young-to-young or old-to-old controls. In contrast, old tissues transplanted into young hosts exhibited a significant deceleration of mortality-associated transcriptomic features, with changes significantly different from those seen in old-to-old and young-to-young groups.

To delineate shared and distinct biological pathways underlying the transcriptomic responses to transplantation, we applied module-specific rodent multi-tissue transcriptomic clocks of chronological age and mortality, which were designed to predict tAge at the level of individual cellular subsystems (modules) [17]. Interestingly, we did not detect any modules that showed significant rejuvenation or aging in transplantation scenarios involving old graft tissue (Figure 1K). In contrast, transplantation involving young grafts, regardless of recipient age, was associated with a significant pro-aging signal according to multiple functional modules. Modules that exhibited the strongest tAge acceleration for both young-to-young and young-to-old transplantations, supported by both chronological and mortality clocks, included adaptive immunity/T cell signaling, innate immunity/inflammation, cholesterol metabolism/platelet degranulation, and respiration/mitochondrial translation. These results suggest that these metabolic and immune domains are particularly sensitive to stress following the transplantation of young adipose tissue. Of note, the chromatin modification (2) module showed an aging effect uniquely in young-to-old transplants, across both types of clocks, which may partially explain the more pronounced epigenetic remodeling observed in this type of transplantation. Finally, certain modules showed aging exclusively during the young-to-young transplantation, specifically lipid metabolism/PPAR signaling, OxPhos/TCA cycle, and apoptosis/Nrf2 signaling/proteasome modules.

### Transcriptomic signatures underlying adipose tissue rejuvenation indicate activation of known and novel thermogenic pathways

To gain functional insight into the molecular changes associated with aging and rejuvenation, we analyzed transcriptional responses within grafts across transplantation conditions (Supplementary Figure 2A). All transplantations, especially those involving either a young graft or a young recipient, elicited numerous gene expression changes in the grafted adipose tissue. The most extensive response was observed in old-to-young transplants, with over 2,000 genes (p.adjusted<0.05) significantly up- or downregulated following 10 weeks of implantation into the young environment. In contrast, old-to-old transplants showed the smallest transcriptomic shift, with only a few hundred differentially expressed genes, which may be partially explained by the lower sample size for this transplantation group.

**Figure 2.**
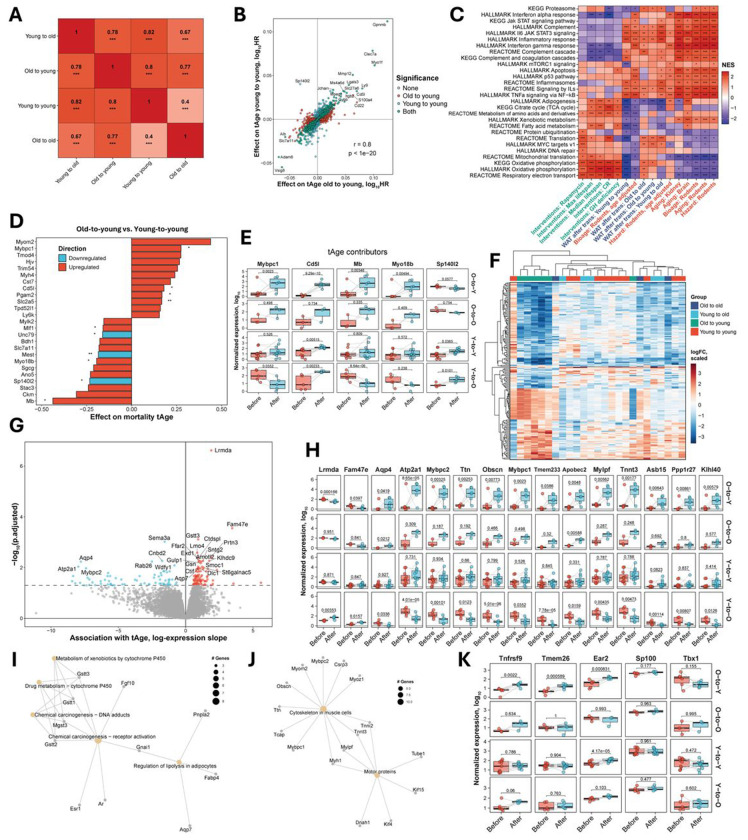
Transcriptomic signatures underlying adipose tissue rejuvenation indicate activation of known and novel thermogenic pathways **(A)** Pearson’s correlations of gene expression changes in WAT samples after transplantation across all experimental groups. Red indicates positive correlation, and the number indicates the correlation coefficient (R). Asterisks reflect statistical significance (BH-adjusted p-values). **(B)** Gene expression changes induced by transplantation in a donor’s WAT that contribute to the change in transcriptomic age for young-to-young and old-to-young groups. R indicates the correlation coefficient, and p indicates the statistical significance (p-value) of the observed correlation. **(C)** Pathway enrichment analysis (GSEA) of gene expression changes induced by transplantation in a donor’s WAT across all experimental groups (darkblue), along with the established signatures of lifespan-extending interventions (green), aging, and mortality in rodents (red). The direction of expression change is reflected with color, and asterisks reflect statistical significance (BH-adjusted p-values). **(D)** Gene contribution to increased and decreased tAge following transplantation in old-to-young vs young-to-young group, according to the Elastic Net (EN) rodent multi-tissue Transcriptomic Mortality Clock. Color and asterisks denote direction and statistical significance (BH-adjusted p-value) of difference in expression dynamics between groups, respectively. **(E)** Normalized expression of top genes, contributing to increased and decreased tAge following transplantation in old-to-young vs young-to-young group, in WAT samples before and after transplantation across all transplantation models. **(F)** Hierarchical clustering of genes, whose expression changes are significantly associated with tAge changes across all transplantation groups. The direction of expression change is reflected with color. **(G)** Volcano plot displaying genes, whose upregulation during transplantation in a donor’s WAT is associated with higher (red) or lower (blue) tAge across all transplantation conditions. **(H)** Top 15 genes, whose normalized expression changes (in log scale) are most significantly associated with tAge changes following the transplantation across all experimental groups, ranked by statistical significance (BH-adjusted p-value). **(I-J)** Pathway enrichment for genes positively **(I)** and negatively **(J)** associated with tAge dynamics during transplantation. Grey nodes represent individual genes, and yellow nodes represent enriched pathways. Yellow node size reflects the number of significantly associated genes in each pathway, and edges indicate semantic similarity between terms. Only statistically significant terms (BH-adjusted p < 0.05, based on Fisher’s exact test) are shown. **(K)** Normalized expression dynamics (in log scale) of established beige adipose tissue-specific markers in WAT samples before and after transplantation across all experimental groups. Boxplots: center line indicates median; box limits, interquartile range; whiskers, ±1.5× IQR. Unless specified otherwise, statistical differences between groups are assessed with ANOVA and adjusted for multiple comparisons with the Benjamini-Hochberg approach. *** p.adjusted< 0.001, ** p.adjusted < 0.01, * p.adjusted < 0.05, ^ p.adjusted < 0.1.

To assess the top contributors of rejuvenation, we focused on deconvolution of Transcriptomic Mortality Clock, as it accurately captures gene-expression patterns linked to lifespan and health outcomes and is therefore best-positioned to identify molecular programs that may counteract aging. Across Mortality Clock genes, we observed all four regulatory patterns: some increased expected hazard when upregulated, others decreased expected hazard when upregulated; conversely, some reduced expected hazard when downregulated, whereas others increased it. Therefore, rejuvenation cannot be attributed to uniform up- or downregulation but rather to a gene-specific rebalancing (Supplementary Figure 2B). Notably, overall changes observed in old adipose tissue transplanted into young recipients were highly and significantly correlated (Pearson’s R > 0.77 for all transplantation groups) with those from all other transplant conditions (Figure 2A). The weakest correlation (Pearson’s R = 0.4) was observed between the isochronic groups (young-to-young vs. old-to-old).

A closer examination of mortality-informative genes revealed an extensive overlap between young-to-young and old-to-young groups, with the same genes increasing and decreasing transcriptomic age in both groups, yielding a strong correlation in expression changes (Pearson’s R = 0.8; Figure 2B). A similar relationship was observed between young-to-old and old-to-young groups (Supplementary Figure 2C).

To further contextualize the transcriptional responses, we compared gene expression changes in grafted tissues following the transplantation against established gene expression signatures associated with aging and mortality across different organs and species, as well as against lifespan-extending interventions in mice [27, 28] (Supplementary Figure 2D). Across all groups, we observed highly significant positive correlations with aging and mortality signatures and negative correlations with intervention signatures, though the magnitude and composition of these associations varied. Among all conditions, the young-to-young group exhibited the most deleterious profile, showing strong and highly significant positive correlations with all aging and mortality signatures, and robust negative correlations with gene expression profiles induced by lifespan-extending interventions. On the other hand, other transplant groups negatively correlated only with calorie restriction gene expression signatures and displayed fewer correlations with other aging and mortality signatures. The old-to-old group appeared the least detrimental, even exhibiting a positive correlation with maximum lifespan extension.

To understand the biological pathways underlying these correlations, we performed a functional enrichment analysis of differentially expressed genes across transplant groups (Figure 2C). All groups displayed upregulation of inflammatory and immune pathways, such as interleukin signaling, general inflammatory response, IL6–JAK–STAT3 signaling, and complement cascade - established hallmarks of aging and mortality in mice. This shared response likely reflects immune rejection triggered by transplantation of exogenous tissue. All transplant types showed downregulation of adipogenesis, a pattern consistent with gene expression signatures of aging and opposite to the one induced by lifespan-extending interventions. The young-to-young group showed the strongest aging signature, marked by pronounced suppression of metabolic and energy pathways, including the TCA cycle, fatty acid metabolism, mitochondrial translation, oxidative phosphorylation, and respiratory electron transport, in line with predictions of module-specific clocks. The young-to-old group also exhibited signs of energy metabolism suppression, in the form of the TCA cycle downregulation. Oxidative phosphorylation was upregulated in both old-to-young and old-to-old groups. Rejuvenation in old-to-young transplants was further marked by upregulation of pro-longevity pathways, including MYC targets and translation, alongside the weakest inflammatory signaling among the groups. In contrast, old-to-old grafts showed similar MYC and translation activation but with broader inflammatory pathway engagement.

Next, we performed a clustering analysis of gene expression changes for the genes across all transplantation conditions. The strongest transcriptomic divergence was observed between old-to-young and young-to-young groups (Supplementary Figure 2E), consistent with the largest number of differentially expressed genes identified between these two conditions (>2,000 genes with p.adjusted < 0.05; Supplementary Figure 2F). In contrast, comparisons involving old-to-young versus old-to-old, and young-to-old versus old-to-old, yielded relatively few differentially expressed genes.

To understand why a young systemic environment rejuvenates aged adipose tissue but accelerates aging in young grafts, we compared young-to-young and old-to-young groups to identify the gene changes contributing to transcriptomic clock predictions across these groups. The most consistent contributions to higher tAge predictions in old-to-young group compared to young-to-young animals according to both Chronological (Supplementary Figure 2G) and Mortality (Figure 2D) Clocks were made by upregulated *Mybpc1* (Myosin Binding Protein-C) and *Cd5l* (CD5 antigen-like), while the most consistent contributions to decreased tAge in old-to-young group were made by upregulated *Mb* (myoglobin) and *Myo18b* (myosin XVIIIb), and by downregulated *Sp140l2* (Sp140 nuclear body protein like 2).

Next, we examined the expression dynamics of the top contributing genes across all transplantation conditions (Figure 2E). In old adipose tissues implanted into young recipients, all genes, except for *Sp140l2*, demonstrated a significant upregulation relative to the baseline levels. Although the decrease in *Sp140l2* expression was only marginally significant, its downregulation contributed to the reduced tAge, alongside upregulation of *Mb* and *Myo18b*. Conversely, upregulation of *Mybpc1* and *Cd5l* increased tAge in this context. *Mybpc1* was significantly downregulated in young grafts placed into old hosts, contributing to a decrease in predicted age, whereas its expression remained unchanged in both isochronic transplantation groups. *Cd5l* was significantly upregulated in all groups except old-to-old, suggesting that macrophage recruitment [29–31] to the graft requires at least one young component, either the graft or the host environment. *Mb* expression was significantly upregulated in old adipose tissue transplanted into young hosts, contributing to the observed reduction in tAge. In contrast, *Mb* expression declined in young grafts placed into aged hosts, aligning with the increase in tAge, and its levels remained unchanged in isochronic transplantation groups. *Myo18b* expression changed exclusively in the old-to-young group, while *Sp140l2* was upregulated in young tissues transplanted into either young or old hosts, contributing to increased tAges for these groups. Interestingly, *Mybpc1*, *Mb*, and *Myo18b*, all muscle-related genes [32], exhibited coordinated regulation across samples, with expression changes occurring in the same direction and magnitude for all samples. Moreover, the directionality of changes for these genes was opposite between old-to-young and young-to-old conditions and absent in isochronic comparisons, suggesting that these genes may be specifically linked to rejuvenation or aging of adipose tissue rather than to general wound healing or graft stabilization.

To identify genes associated with adipose tissue rejuvenation, we performed an unbiased transcriptomic screen by incorporating all experimental groups into a joint analysis. Specifically, we searched for genes whose expression changes correlated with changes in predicted expected hazard values derived from the Mortality Transcriptomic Clock. In accordance with clock predictions, changes induced by old-to-young transplantation clustered separately from those induced by other modes of transplantation (Figure 2F). In total, we identified 172 genes (p.adjusted < 0.05) whose expression changes significantly correlated with the changes in biological age (Figure 2G). The top 15 genes included *Lrmda*, *Fam47e*, *Aqp4*, *Atp2a1*, *Mybpc2*, *Ttn*, *Obscn*, *Mybpc1*, *Tmem233*, *Apobec2*, *Mylpf*, *Tnnt3*, *Asb15*, *Ppp1r27*, *Klh40*. Most of these genes were previously linked to improved adipose tissue health through reductions in lipid droplet size due to modulation of thermogenic pathways (see Discussion for details).

Inspection of both clustering and gene expression dynamics (Figures 2F and 2H) revealed coordinated changes among sets of genes, similar to the ones observed for *Mybpc1*, *Mb*, and *Myo18b* in previous analysis. These genes changed in opposite directions in old-to-young versus young-to-old transplantations, while showing minimal change in the isochronic transplantations (old-to-old and young-to-young) (Figure 2H). This suggests that these genes are specifically involved in age-related remodeling of adipose tissue, rather than general tissue repair following transplantation. To determine whether these rejuvenation-associated genes shared common biological functions, we performed a pathway enrichment analysis. Genes downregulated during rejuvenation were significantly enriched for pathways associated with chemical carcinogenesis, metabolism of xenobiotics by cytochrome P450, and regulation of lipolysis in adipocytes pathways (Figure 2I). Many of the genes related to cytochrome P450 and chemical carcinogenesis were glutathione transferases (*Gstt1/ Gstt2/Gstt3* (Glutathione S-Transferase Theta 1/2/3), and *Mgst3* (Microsomal Glutathione S-Transferase 3)), involved in cellular detoxification and protection from oxidative damage, particularly lipid peroxidation in adipocytes [33–35]. Among the lipolysis-related genes downregulated during rejuvenation were *Aqp7*, *Fabp4*, and *Pnpla2*. Finally, downregulated genes associated with rejuvenation included *Gnai1*, which connected chemical carcinogenesis and regulation of lipolysis pathways. In contrast, genes upregulated during rejuvenation were enriched for cytoskeletal and motor protein pathways commonly expressed in muscle cells.

To determine whether established thermogenesis markers were altered in aged adipose tissue following transplantation into young recipients, we first examined expression changes of well-characterized brown and beige adipocyte thermogenic markers, including *Ebf2*, *Prdm16*, *Ppara*, *Cidea*, *Dio2*, *Cox8b*, *Cox7a*, *Ucp1*, and *Atp2a2* [36–40] (Supplementary Figure 2H). *Ebf2* and *Prdm16*, critical transcriptional regulators of beige adipocyte identity [39], were significantly suppressed in both old-to-young and young-to-old transplantations, while remaining stable in isochronic conditions. *Ppara* was significantly downregulated only in the old-to-young group. *Cidea* levels decreased in every group except the old-to-old group. *Dio2* levels increased in the young-to-old group, with no significant changes in every other transplantation group. *Cox8b* was significantly decreased in young grafts regardless of recipient ages, and *Cox7a1* remained unchanged across conditions. Finally, neither *Ucp1*, the most well-known marker of thermogenic activation, nor *Atp2a2*, encoding a calcium pump involved in UCP1-independent thermogenesis, were significantly altered in any transplantation scenario. Together, these findings indicate suppression of the canonical thermogenic program, mainly related to brown adipose tissue (BAT), in both heterochronic transplantation contexts. If changes in thermogenesis occur during rejuvenation of old adipose tissue in young hosts, they likely proceed through a non-canonical, previously uncharacterized thermogenic remodeling pathway.

To further explore the possibility that rejuvenation engages thermogenic programs, we assessed the expression of established beige adipocyte-specific markers (Figure 2K). *Tnfrsf9* (also frequently reported as CD137) and *Tmem26* - the most robust and validated markers of beige adipocytes [37] were markedly and exclusively upregulated in the old-to-young group, providing strong molecular evidence for beige phenotype induction upon rejuvenation. We further evaluated *Ear2*, *Sp100*, and *Tbx1*, a subset of beige-specific genes proposed to be activated in response to *Ebf2* downregulation [39]. *Ear2* was significantly upregulated in both old-to-young and young-to-young groups, whereas *Sp100* and *Tbx1* showed no significant changes in any transplantation condition. These findings suggest that while expression levels of canonical thermogenic drivers such as *Ucp1* and *Atp2a2* remain unaltered, rejuvenation is accompanied by partial engagement of known beige adipocyte-specific gene programs.

### Histological characterization reveals lipid droplet size as a central feature of adipose tissue rejuvenation

**Figure 3.**
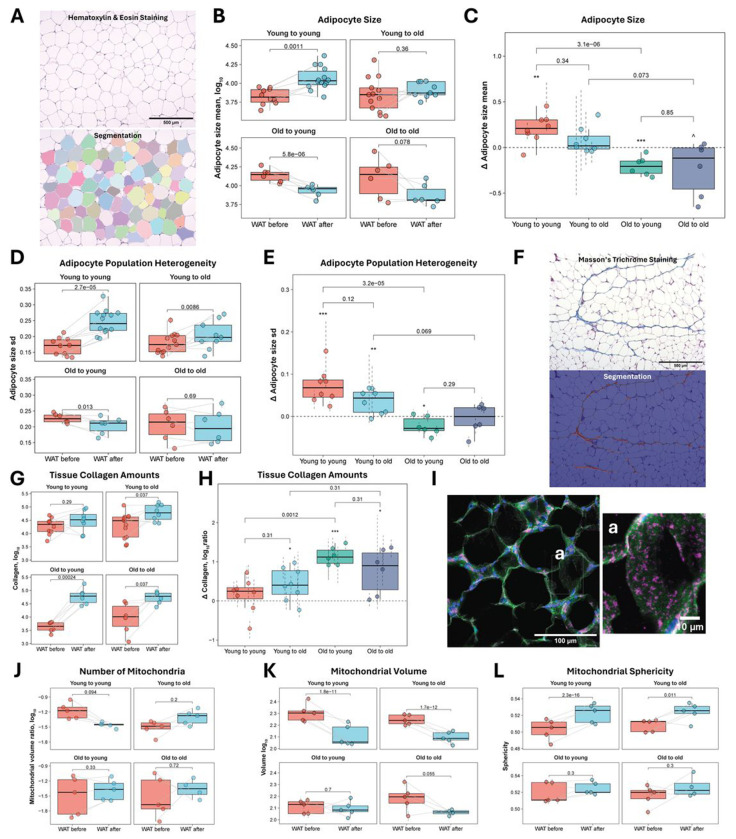
Histological characterization reveals lipid droplet size as a central feature of adipose tissue rejuvenation. **(A)** Example hematoxylin and eosin (H&E)-stained section of inguinal white adipose tissue (top) and selected adipocytes used for lipid droplet size quantification (bottom). **(B)** Quantification of adipocyte lipid droplet size ratio (in log scale) in WAT samples before and after transplantation across all experimental groups. Mouse ID was included in the statistical model as a covariate to ensure paired comparison of lipid droplet size ratios. Differences in mean droplet size before and after transplantation were assessed with mixed-effects models. Data are mean droplet size (in log scale) ± s.e.m. **(C)** Comparison of adipocyte lipid droplet size (in log scale) dynamics in WAT samples before and after transplantation across all experimental groups. Asterisks reflect statistical significance (BH-adjusted p-values) of lipid droplet size changes during transplantation within individual groups, whereas significance of pairwise comparisons in lipid droplet size ratio dynamics between groups is denoted in text. Differences in mean droplet size changes were assessed with mixed-effects models. Data are mean droplet size Δ (in log scale) ± s.e.m **(D)** Quantification of adipocyte lipid droplet size (in log scale) standard deviation in all WAT samples before and after transplantation across all experimental groups. Mouse ID was included in the statistical model as a covariate to ensure paired comparison of lipid droplet size standard deviation ratios. Differences in droplet size standard deviation before and after transplantation were assessed with mixed-effects models. Data are mean standard deviation of adipocyte size (in log scale) ± s.e.m. **(E)** Comparison of lipid droplet size (in log scale) standard deviation dynamics in WAT samples before and after transplantation across all experimental groups. Differences in droplet size standard deviation changes were assessed with mixed-effects models. Asterisks reflect statistical significance (BH-adjusted p-values) of lipid droplet size standard deviation changes during transplantation within individual groups, whereas significance of pairwise comparisons in lipid droplet size standard deviation ratio dynamics between groups is denoted in text. Data are mean standard deviation Δ ± s.e.m. **(F)** Example image of adipose tissue stained with Masson’s Trichrome (top) and computationally segmented collagen-positive regions used for quantification (bottom). **(G)** Quantification of collagen deposition ratio (in log scale) in WAT samples before and after transplantation across all experimental groups. Mouse ID was included in the statistical model as a covariate to ensure paired comparison of collagen deposition ratios. **(H)** Comparison of collagen deposition ratio (in log scale) dynamics in WAT samples before and after transplantation across all experimental groups. Differences in collage deposition changes across groups were assessed with mixed-effects models. Asterisks reflect statistical significance (BH-adjusted p-values) of collagen deposition changes during transplantation within individual groups, whereas significance of pairwise comparisons in collagen deposition ratio dynamics between groups is denoted in text. Data are mean collagen amount (in log scale) Δ ± s.e.m. **(I)** Immunofluorescent staining of 15 μm adipose tissue sections for mitochondria (Tomm20, magenta), adipocyte cell membrane (autofluorescence, green) and nuclei (DAPI, blue); panel (a) shows higher magnification highlighting mitochondrial labeling. **(J)** Quantification of mitochondrial density ratio (number per cytoplasmic volume, in log scale) in all WAT samples before and after transplantation across all experimental groups. Mouse ID was included in the statistical model as a covariate to ensure paired comparison of mitochondrial density ratios. **(K)** Quantification of mitochondrial volume (in log scale) in WAT samples before and after transplantation across all experimental groups. Mouse ID was included in the statistical model as a covariate to ensure paired comparison of mitochondrial volume ratios. Changes of mitochondrial volume following transplantation were assessed with mixed-effects models. Data are mean mitochondrial volume (in log scale) ± s.e.m. **(L)** Quantification of mitochondrial sphericity in WAT samples before and after transplantation across all experimental groups. Mouse ID was included in the statistical model as a covariate to ensure paired comparison of mitochondrial sphericities. Changes of mitochondrial sphericity following transplantation were assessed with mixed-effects models. Data are mean ± s.e.m. Boxplots: center line indicates median; box limits, interquartile range; whiskers, ±1.5× IQR. Unless specified otherwise, statistical differences between groups are assessed with ANOVA and adjusted for multiple comparisons with the Benjamini-Hochberg approach. *** p.adjusted< 0.001, ** p.adjusted < 0.01, * p.adjusted < 0.05.

Given that the rejuvenation-associated genes identified in old adipose tissue grafts placed in young recipients were highly involved in lipid droplet size regulation and activation of thermogenesis, we decided to assess adipocyte lipid droplet sizes across transplantation groups. To evaluate whether the aging and rejuvenation dynamics observed with epigenetic and transcriptomic clocks are reflected in adipocyte morphology, we performed Segment Anything Model (SAM)-based quantification of lipid droplet sizes from Hematoxylin and eosin (H&E)-stained sections of WAT collected before and after transplantation (Figure 3A). Consistent with prior reports that aging is associated with adipocyte hypertrophy, baseline measurements showed that old WAT generally exhibited larger lipid droplets compared to young WAT, although some variability was present (Figure 3B). Following transplantation, we observed an increase in lipid droplet size in young adipose tissue implanted into young recipients, and conversely, a significant reduction in droplet size in old adipose tissue implanted into young hosts. At the same time, young adipose tissues implanted into old recipients did not exhibit a significant increase in droplet size, and old adipose tissues implanted into old recipients exhibited a marginally significant decrease in lipid droplet size. Comparison of lipid droplet size dynamics across transplantation groups revealed that old adipose tissues transplanted into young hosts underwent significantly stronger reduction in droplet size compared to young tissues transplanted into young hosts, but not relative to old tissues transplanted into old hosts (Figure 3C). Young adipose tissue grafts implanted into either young or old hosts exhibited similar degrees of change, although those placed into old hosts showed a marginally greater increase in lipid droplet size compared to old grafts transplanted into old recipients.

We next quantified intra-tissue heterogeneity in adipocyte size, using the standard deviation of lipid droplet area (in log scale) as a proxy. Overall, variation in adipocyte size mirrored the patterns observed for mean lipid droplet size. Thus, young grafts in young recipients exhibited increased heterogeneity (Figure 3D), consistent with expansion of the largest adipocyte subpopulations and a possible shift toward a hypertrophic phenotype. In contrast, old adipose tissues transplanted into young hosts showed a marked reduction in heterogeneity, indicating not only a global reduction in adipocyte size but also its stabilization across the population. Young adipose tissue transplanted into old recipients displayed increased size heterogeneity, suggesting the emergence of hypertrophic subpopulations even in the absence of significant mean size change. Lastly, the old-to-old group showed no changes in heterogeneity. Comparison of adipocyte heterogeneity change trajectories revealed that old adipose tissues transplanted into young hosts underwent significantly different dynamics compared to young tissues transplanted into young hosts, but not relative to old tissues transplanted into old hosts (Figure 3E). Young adipose grafts implanted into either young or old hosts exhibited similar degrees of change, although those placed into old hosts showed a marginally greater increase in adipocyte heterogeneity compared to old grafts transplanted into old recipients.

To investigate macrophage infiltration suggested by elevation of *Cd5l* levels in all groups except the old-to-old scenario, we examined the expression dynamics of immune and adipocyte lineage markers. Specifically, we assessed *Cd68* (pan-macrophage marker) [41], *Mrc1* (M2-activated anti-inflammatory macrophages) [42], *Cd8* (cytotoxic T-cell marker) [43], and the adipocyte marker *Pparg* (Peroxisome proliferator-activated receptor gamma) [44] to infer shifts in cellular composition following transplantation (Supplementary Figure 3A). We hypothesized that increased *Cd68* and *Cd8a* expression, coupled with reduced *Pparg* levels, would indicate heightened immune cell infiltration and a relative decline in adipocyte-specific signals within bulk RNA-seq profiles. Consistent with the *Cd5l* findings, *Cd68* expression was significantly upregulated in all transplantation conditions except old-to-old, confirming robust macrophage infiltration when either the graft or recipient was young. *Mrc1* levels were selectively elevated in the old-to-young and young-to-young groups, reflecting enhanced recruitment of anti-inflammatory macrophages in young environments. *Cd8a* was significantly upregulated only in young grafts implanted into old recipients, indicating increased cytotoxic T-cell infiltration, potentially contributing to the observed rapid aging of the young graft. Finally, *Pparg* expression was significantly reduced in both old-to-young and young-to-old conditions, suggesting the mitigation of the signal from adipocytes for these groups during bulk adipose tissue sequencing.

To characterize tissue remodeling following transplantation, we evaluated collagen deposition in WAT using Masson’s Trichrome staining (Figure 3F). Elevated collagen levels have been linked to macrophage recruitment and adipose pathologies such as diet-induced obesity, hypoxia, and fibrosis [45–48]. Post-transplantation, collagen levels increased significantly across all groups except the young-to-young controls, with endpoint levels converging across transplantation types (Figure 3G). The largest increase in collagen content was observed in old grafts implanted into young hosts, and the trajectory analyses revealed that old-to-young transplants exhibited significantly different collagen accumulation dynamics compared to young-to-young transplants, but not relative to old-to-old grafts (Figure 3H). In contrast, young grafts implanted into either young or old recipients showed comparable changes, and young-to-old transplants did not change significantly differently from old-to-old controls. The analysis of collagen content revealed that young tissues contained higher baseline levels of collagen than old tissues prior to transplantation, suggesting that collagen accumulation during fibrosis may reflect adaptive remodeling rather than degenerative changes. Alternatively, the apparent increase could result from enhanced visibility of collagen in regions with smaller or less densely packed adipocytes. Consistent with collagen staining, fibrosis increased post-transplantation in all groups, but changes were not significantly different between any of the compared transplantation types (Supplementary Figure 3C). Notably, the strongest fibrotic response occurred in young-to-old grafts, while old-to-young grafts showed the least increase. These findings suggest that transplantation broadly activates fibrotic remodeling, with host age exerting a modest influence on its extent.

We next examined whether mitochondrial abundance and morphology were altered in WAT following transplantation. Mitochondrial fragmentation and increased sphericity are established features of aging [49], although recent studies suggest that increased fragmentation can be uncoupled from aging, potentially representing changes that are neither adaptive nor deleterious, but instead reflect alternative cellular processes [50]. 15 μm-thick tissue sections used for quantitative analysis (Figure 3I) revealed that old and young tissues before transplantation had comparable numbers of mitochondria, and mitochondrial abundance did not significantly change post-transplantation across any transplantation groups, regardless of donor or host age (Figure 3J). Mitochondria in young adipose tissues on average exhibited greater volumes and lower sphericity, consistent with prior observations of more elongated and connected mitochondrial networks in young cells. Upon transplantation into either young or old hosts, young tissues displayed reduced mitochondrial volume (Figure 3K) and increased sphericity (Figure 3L), indicative of enhanced mitochondrial fragmentation. In contrast, old adipose tissues transplanted into young or old recipients showed no significant changes in mitochondrial volume or shape, suggesting that age-related changes in mitochondrial morphology are resistant to rejuvenation. These findings imply that only select age-related features, potentially those most relevant to function, are reset by transplantation, while others may remain unaffected, at least within the timeframe of the conducted study.

## Discussion

Across both epigenetic and transcriptomic biomarkers of aging, whether trained on chronological age or mortality risk, our transplantation experiments reveal that the aging trajectories of grafted adipose tissues were determined by the recipient’s age and the baseline age of the donor tissue. Epigenetic and transcriptomic clocks showed that old adipose tissues grafted into young recipients became younger in their molecular features, whereas young adipose tissues placed in old recipients rapidly aged, which closely mirror classic parabiosis paradigms and support a model in which youthful systemic environments can reverse molecular features of aging in old tissues. Notably, rejuvenated grafts did not fully reach the age of young hosts, likely due to procedural pro-aging effects associated with immune response observed even in isochronic transplantations. Elevated epigenetic age in isochronic settings suggests that the transplantation itself induces measurable aging, driven by inflammatory responses, immune cell infiltration, and reparative processes. Transcriptomic correlations across all experimental groups further confirm the presence of a shared injury response program, likely encompassing implantation, vascularization, healing, and immune activation, confounding the interpretation of host-specific effects. Despite this shared graft survival response, old adipose tissues transplanted into young hosts still underwent distinct and measurable rejuvenation. Targeting this confounding process of graft implantation, for example, with pharmacological dampening of post-transplant inflammation, may help mitigate the pro-aging effects of young-to-old grafting and amplify the rejuvenation observed in old-to-young contexts.

Module clocks and transcriptomic clock gene contribution analyses revealed that aging and rejuvenation are not likely to be impacted by a single gene or pathway but instead reflect a complex and multidimensional remodeling process. Tissue age reversal is likely not absolute or uniformly directional but rather represents a multifaceted and context-dependent rebalancing of multiple cellular and molecular features. Analysis of genes that contributed to increased and decreased transcriptomic ages when comparing young-to-young versus old-to-young or young-to-old versus old-to-young groups again suggests that the young systemic environment induces a broadly similar transcriptional response across grafts, regardless of donor age; yet, the biological age outcome diverges depending on the baseline age of the implanted tissue. Among the shared genes contributing to increased transcriptomic age, *Gpnmb*, a well-characterized pro-inflammatory factor associated with aging and mortality in both mice and humans [17], was the top contributor. In contrast, *Vsig8*, an immunosuppressive regulator of T cell function previously linked to pro-tumorigenic activity [51], emerged as the top contributor to a transcriptomic age reduction in all groups. This finding aligns with prior work suggesting that cancer and rejuvenation may involve overlapping molecular programs, particularly those activated during cellular reprogramming [52]. Another top rejuvenation-associated gene, *Adam8*, encodes a metalloprotease involved in inflammation, tissue remodeling, and cancer progression [53, 54], further supporting a shared mechanistic axis between rejuvenation, regeneration, and oncogenesis.

The analysis of genes distinguishing rejuvenation from aging within the young systemic environment revealed interesting gene expression patterns in how the young body reprograms the energy metabolism of the old graft. While *Mybpc1* is best known for its role in muscle contractility [55], recent evidence suggests a role in thermogenic regulation within BAT [56]. The strongest transcriptomic age decrease-associated gene, Myoglobin (*Mb*), classically characterized as a muscle-specific oxygen-binding protein, has recently been found to be an important player in intracellular lipid delivery to mitochondria of brown and likely beige (or brite) adipocytes [57–60]. Specifically, it was shown that higher expression of myoglobin results in a more effective energy metabolism and smaller lipid droplet sizes due to enhanced thermogenesis. The selective upregulation of *Mb* and *Mybpc1* in old adipose tissue transplanted into young hosts suggests that rejuvenation is linked to activation of thermogenic capacity in aged adipocytes. This process may be further facilitated by the macrophage-derived AIM, as indicated by the concurrent upregulation of *Cd5l*. *Cd5l* from macrophages that infiltrate the tissue was shown to directly regulate the lipid droplet size in adipocytes by inhibiting the fatty acid synthase activity and thereby activating the lipolysis and reducing the adipocyte sizes. However, this happens at the cost of increased inflammation, macrophage infiltration, development of insulin resistance, and glucose intolerance [29–31]. In contrast, rapid aging of young grafts in aged environments appears linked to suppression of the myogenic gene-related thermogenic program. Notably, the expected increase in lipid droplet size may be partially mitigated by the increased presence of AIM in the young-to-old tissues following the transplantation. As for the upregulation of *Myo18b*, and downregulation of *Sp140l2*, which were also significantly associated with decrease of transcriptomic age, we could not find the data in the literature about the role of these genes in adipocyte metabolism. However, this may imply that *Sp140l2*, and especially *Myo18b* may be previously unidentified players in adipocyte thermogenesis and lipid droplet size regulation. Altogether, the comparison of gene expression changes between young-to-young and old-to-young types of transplantation allowed us to hypothesize that old adipose tissue transplanted into young animals undergoes macrophage infiltration, activation of thermogenic pathways, initiation of a beiging program, and a consequent reduction in lipid droplet size.

Our transcriptomic screen to identify genes whose expression changes correlated with changes in predicted biological age revealed that the most significantly downregulated gene associated with rejuvenation was *Lrmda* (leucine-rich melanocyte differentiation associated). *Lrmda* has previously been linked to increased creatinine levels and improved grip strength in knockout models [61]. Elevated creatinine may reflect higher levels of creatine [62, 63], a metabolite whose supplementation has been shown to protect against high-fat diet-induced obesity, enhance insulin sensitivity, improve glucose homeostasis, and promote lipolysis, lipophagy, and thermogenesis in BAT [64]. Another significantly downregulated gene in rejuvenated tissues, *Fam47e* (family with sequence similarity 47, member E), was previously studied in biopsies of human subcutaneous adipose tissue, where lower expression levels were associated with reduced waist circumference, fasting insulin, and HOMA-B index after adjusting for BMI and cell-type proportions [65]. The directionality of these gene expression changes again suggests enhanced energy metabolism due to the activation of a thermogenic program, which should result in a reduction in adipocyte hypertrophy and lipid droplet size.

*Aqp4* (aquaporin-4), which was strongly upregulated during rejuvenation but downregulated during aging, encodes a key regulator of osmotic and calcium homeostasis [66]. Its expression declines with age in skeletal muscle, and we similarly observed lower levels in aged adipose tissues prior to transplantation. Its robust upregulation in rejuvenated tissue may reflect restoration of homeostatic capacity. *Atp2a1* (SERCA1), another gene markedly upregulated during rejuvenation and downregulated during rapid aging, encodes a calcium pump responsible for the maintenance of cellular homeostasis by transferring cytosolic calcium into the endoplasmic reticulum in an ATP-dependent manner [67]. At the same time, the process of futile calcium transfer generates heat through ATP hydrolysis and has been implicated in muscle thermogenesis [68]. Relatedly, *Atp2a2* (SERCA2b) has been identified as a UCP1-independent driver of white adipocyte thermogenesis and beiging, acting through a similar mechanism of futile calcium transport [69]. Other top genes upregulated during rejuvenation, including *Mybpc2*, *Ttn*, *Obscn*, *Mybpc1*, *Tmem233*, *Apobec2*, *Mylpf*, *Tnnt3*, *Asb15*, *Ppp1r27*, *Klhl40*, were previously related to the muscle tissue [32]. At the same time, every gene in this list except *Asb15*, *Ppp1r27*, and *Klh40* was found to play a role in the biology of BAT [56]. Moreover, expression of several muscle-associated transcripts, including *Atp2a1*, *Tnnt3*, *Ttn*, *Mybpc2*, and *Mylpf* (full list of genes for the comparison is not available elsewhere) [70], has been shown to decline in subcutaneous adipose tissue of mice fed a high-fat diet and exhibiting the most pronounced insulin resistance. Individual genes from this set, such as *Ttn* (Titin), have previously been implicated in the maintenance of adipose tissue thermogenic capacity. *Ttn* expression markedly declines with age in BAT [71], is significantly downregulated in the brown fat of obesity-prone rats [72], and mutations in *Ttn* lead to severe thermoregulatory impairment due to disrupted brown adipose tissue function [73]. The functional coordinated role of these myogenic genes in adipose tissue likely centers on generating tensional responses and uncoupling of calcium transport from ATP production, thereby facilitating the utilization of the stored energy and heat generation [74]. In particular, we speculate that the muscle genes upregulated during rejuvenation may parallel the function of *Ryr1* and *Ryr2* in mediating SERCA-mediated futile calcium flux resulting in heat production in skeletal muscle and beige adipocytes, respectively [68, 69]. Downregulation of these genes may be a hallmark of metabolically compromised adipose tissue, whereas their upregulation during rejuvenation may reflect improved metabolic competence and the reactivation of thermogenic potential.

We further observed that rejuvenation was associated with downregulation of detoxification and cellular protection pathways - a pattern that may reflect either diminished physiological stress under improved systemic conditions or reduced protective capacity; the biological significance of this shift remains to be determined. Among the lipolysis-related genes downregulated during rejuvenation were *Aqp7*, *Fabp4*, and *Pnpla2*. *Aqp7* (aquaporin 7) facilitates glycerol efflux following lipolysis, and its deficiency leads to intracellular glycerol accumulation, impaired lipolysis, and adipocyte hypertrophy [75, 76]. *Fabp4* (fatty acid-binding protein 4) is a fatty acid transporter whose elevated levels have been associated with multiple age-related metabolic and cardiovascular disorders [77]. *Pnpla2* (adipose triglyceride lipase (ATGL)) initiates the first step of triglyceride hydrolysis and is a rate-limiting regulator of lipolysis; its deficiency results in lipid accumulation in hearts, obesity, cardiometabolic dysfunction, and impaired thermogenesis [78, 79]. Given the observed reduction in lipid droplet size in old adipose tissue transplanted into young hosts, we speculate that the downregulation of lipolytic genes reflects a diminished demand for lipolysis rather than its deleterious inhibition. Finally, we identified *Gnai1*, a Gαi subunit that inhibits adenylyl cyclase, as significantly downregulated during rejuvenation [80]. *Gnai2*, a closely related paralog, has been shown to regulate adipocyte lipolysis: its deletion enhances β-adrenergic stimulation-induced cAMP accumulation, resulting in enhanced lipolysis, smaller adipocytes, decreased inflammation, reduced weight gain, smaller fat depots in inguinal subcutaneous WAT, improved energy homeostasis, and elevated whole-body energy expenditure compared to controls on high-fat diet [81]. Thus, downregulation of *Gnai1* may similarly enhance cAMP–PGC-1α signaling, promoting lipolysis and activation of thermogenic gene programs ultimately favoring beiging of WAT [82, 83].

Notably, WAT cellular composition likely shifted following transplantation, particularly in heterochronic transplantation groups, with lineage marker profile changes indicating an increased proportion of immune cells and a relative reduction in adipocytes. Consequently, decreased or unchanged expression of adipocyte-specific genes and increased expression of immune-related genes in the bulk RNA-seq data may partially reflect these compositional changes rather than true transcriptional alterations within cell types. As such, any observed increases in adipocyte gene expression may in fact be underestimated. Interestingly, both old and young WAT grafts in young hosts exhibited an increased proportion of M2-activated macrophages. This macrophage polarization pattern has been associated with lower adiposity and improved glycemic control in humans [42], suggesting a healthier response to implantation and a potential reduction in lipid droplet size in these transplants.

Given that many genes associated with adipose tissue rejuvenation are involved in regulating the efficiency of energy metabolism and facilitation of thermogenesis, we reasoned that one of the most direct ways to validate our findings would be to examine a core phenotypic hallmark of adipocyte biology - the size of the lipid droplet. Lipid droplets are evolutionarily conserved organelles that serve as intracellular reservoirs of neutral lipids and play critical roles in maintaining energy, structural, and signaling homeostasis across diverse cell types [84–87]. Mature adipocytes championed in lipid storage function, typically harboring a single (unilocular), large lipid droplet that expands markedly in states of energy excess. In response to insulin, adipocytes mobilize lipids through lipolysis and release non-esterified fatty acids into the blood circulation. However, during aging or in the context of persistent caloric surplus, adipocytes may enter a hypertrophic state characterized by enlarged lipid droplets, loss of thermogenic function, and metabolic dysfunctions such as insulin resistance, obesity, and elevated risk of type 2 diabetes [88–91]. These parallels suggest that obesity itself may represent a state of accelerated adipocyte aging. Under this framework, targeting adipocyte aging, potentially through the induction of a newly identified and/or known thermogenesis program, rather than treating obesity as an isolated metabolic disorder, could theoretically restore youthful adipocyte function and, consequently, alleviate obesity as a downstream outcome.

By contrast, brown and beige adipocytes are characterized by small, often fragmented (multilocular) lipid droplets dispersed throughout the cytoplasm [92]. Individuals with higher levels of brown adipose tissue exhibit a markedly reduced prevalence of cardiometabolic diseases, including cardiovascular diseases, hypertension, and type 2 diabetes [93]. This protective association is underpinned by the unique capacity of thermogenic adipocytes to increase energy expenditure by actively utilizing glucose and lipids - the process that improves systemic metabolic flexibility, reduces lipid burden, and collectively buffers against age- and obesity-associated metabolic decline. The reduction in lipid droplet size observed in old WAT transplanted into young hosts, potentially marking a shift toward the formation of fragmented intracellular droplets, indicates improved adipose tissue health. This morphological rejuvenation aligns with the upregulation of *Mb*, *Cd5l*, *Tnfrsf9*, *Tmem26*, and components of a newly identified *Atp2a1*-driven thermogenic program in WAT. We propose that the observed reduction in droplet size reflects activation of a beiging-like thermogenic program specifically induced by exposure of aged adipose tissue to a young systemic environment. Notably, aged adipose tissues prior to transplantation exhibited higher adipocyte population heterogeneity scores compared to young tissues, suggesting that lipid droplet size heterogeneity may represent a novel aging-associated adipocyte feature and a potential biomarker for assessing adipocyte aging. The dynamics of heterogeneity changes better mirrored the predictions of the aging clocks compared to the dynamics of lipid droplet size changes. This alignment between heterogeneity dynamics and clock predictions indicates that this feature is responsive to interventions aimed at reducing the biological age of adipose tissue. The decrease in adipocyte population heterogeneity following the transplantation of old WAT into the young body could suggest either reversal of hypertrophy in large, dysfunctional adipocytes or selective elimination of the most hypertrophic cells, either outcome aligning with a rejuvenation phenotype. Together, these findings demonstrate that cellular-level structural remodeling, characterized by a reduction in lipid droplet size and a narrowed size distribution across the adipocyte population, is associated with the initiation of the thermogenic program and underlies the rejuvenation observed following the transplantation of old adipose tissue into a young systemic environment.

## Conclusion

We show that subcutaneous WAT can undergo rapid aging or rejuvenation following subcutaneous transplantation, depending on the recipien’s chronological age. Epigenetic and transcriptomic aging clocks revealed directionality in age dynamics, and deconvolution of transcriptomic clocks enabled gene-level characterization of the rejuvenation process. Old WAT transplanted into young hosts exhibited activation of known and previously unrecognized *Atp2a1*-driven WAT thermogenic programs. Histological evaluation of old transplanted tissues validated transcriptomic findings, revealing reduced lipid droplet size and decreased lipid droplet size heterogeneity post-transplantation. Rejuvenated WAT also showed increased collagen deposition, while mitochondrial abundance and morphology remained unchanged, suggesting that the former age-related changes are adaptive, while the latter may be dispensable for the rejuvenation of white adipose tissue. Rejuvenation of white adipose tissue through activation of thermogenic programs may represent a promising strategy to counteract obesity and the accompanying acceleration of metabolic aging, offering a novel route to restore energy balance, improve metabolic flexibility, and potentially slow systemic physiological decline.

### Limitations

This study focused on male subjects of the same genetic background. Opting for the same genetic background was crucial to mitigate potential challenges related to the immunocompatibility of transplanted fat. While these issues could have been addressed with the use of immune suppressants, incorporating them in our study might have introduced a confounding variable. The decision to select only one sex was influenced by the unavailability of old female mice at the time when study was conducted. It would be interesting to replicate the study in females and in genetically heterogeneous mice that more closely resemble the diversity found in human populations. Furthermore, the duration of exposure to the recipient environment is likely to critically influence the magnitude and trajectory of aging or rejuvenation, underscoring the importance of testing additional time points to better characterize the temporal dynamics of adipose tissue aging plasticity. In addition, our findings may be significantly influenced by shifts in cell composition and immune cell infiltration from the recipient, potentially confounding interpretations of cell-level rejuvenation. Likewise, genes associated with rejuvenation may reflect changes in cellular proportions or a signal coming from infiltrated cells rather than intrinsic reprogramming of adipocytes. Dissecting adipocyte-specific biological age and transcriptomic changes at single-cell resolution following transplantation will be critical to determine whether true cellular rejuvenation occurs and to characterize its molecular features. Finally, genes associated with rejuvenation may not play a causal role but instead reflect downstream or parallel processes. Functional validation using targeted perturbation strategies such as genetic knockout or RNA interference will be essential to delineate the mechanistic contributions of these candidate genes and pathways to adipose tissue aging and rejuvenation.

## Methods

### Animals

All experiments were conducted following NIH guidelines and protocols approved by the IACUC at Joslin Diabetes Center (JDC). All experiments were performed using ~3.5- to 4-month-old male C57BL/6 mice purchased from Charles River Laboratories, MA, and ~17.5 to 19-month-old male C57BL/6 mice obtained from the National Institute of Aging aged colony. All mice were housed on a 12/12 h light/dark cycle. Standard mouse chow diet (9F 5020 Lab Diet, PharmaServ, Inc.) and water were provided ad libitum.

### Adipose Tissue Transplantation

All mice were randomly assigned to experimental groups (sham, donor, or recipient) one week prior to surgery. All animals underwent identical preparatory and surgical procedures, differing only in the manipulation of the inguinal white adipose tissue (iWAT): sham animals retained their native fat pads and received no transplants; donors underwent surgical excision of their iWAT pads, and recipients received donor fat implanted subcutaneously in the interscapular region, while their own iWAT remained intact. Surgeries were performed during a fixed 3-hour window to control for circadian variation, with group assignments randomized across surgical days. On the day of surgery, anesthesia was induced with intraperitoneal sodium pentobarbital (75 mg/kg for young and 65 mg/kg for old mice), followed by maintenance with 3.5% isoflurane administered via nose cone. Surgical sites were shaved and sterilized using Betadine. Core body temperature was maintained at 36°C throughout using a heating pad and monitored with a rectal probe. Inguinal fat pads were harvested from donors, weighed, and immediately transferred into 15 mL sterile tubes filled with antibiotic dissolved in PBS and maintained at 37°C in a water bath. Donor incisions were closed using sterile sutures, and mice were allowed to recover on a 37°C heating pad. Recipient mice underwent a dorsal incision over the interscapular region, where donor iWAT was inserted subcutaneously. The amount of implanted tissue was normalized to 1% of the recipient’s body weight to ensure consistent graft load across animals. A portion of donor tissue was retained for histological and omics analyses, with samples for omics being flash-frozen in liquid nitrogen immediately after collection, and samples for histology fixed in 3.5% paraformaldehyde. Sham-operated animals were further randomized to receive surgical incisions at either the donor or the recipient sites. Following surgery, all mice were singly housed for the duration of the study. At study endpoint, the following tissues were collected for analysis after isoflurane-induced anesthesia followed by cardiac puncture to collect blood and plasma followed by removal of vital organs, such as: heart, liver, kidney, visceral white adipose tissue (vWAT), native subcutaneous white adipose tissue (except in donors), transplanted fat (in recipients), triceps, gastrocnemius muscle, cortex, skin at the surgical site, and skin from an unaffected area.

### Isolation of nucleic acids

DNA and RNA were isolated using the Chemagic360 system (Revvity) with the appropriate kits (CMG-723 for tissue DNA; CMG-1212 for tissue RNA). Frozen adipose tissue samples were homogenized in the appropriate lysis buffer from Chemagic360 kits using a Bead Ruptor (Omni International) with 2.8mm ceramic bead filled tubes, using the 2 ml liver pre-set program. The homogenates were centrifuged (18,000 x g, 10 min) to remove insoluble material then processed according to the appropriate Chemagic360 protocol. DNA and RNA was eluted into molecular biology grade water (HyClone HyPure, Cytiva). When necessary, DNA samples were concentrated using a Centrivap (Labconco). Concentration of DNA/RNA samples was determined using Qubit or Quant-it kits (Invitrogen). Isolated DNA was stored at −20°C and isolated RNA was stored at −80°C until further processing.

### DNA methylation profiling

Methylation data was generated through the Epigenetic Clock Development Foundation using the HorvathMammalMethyl40 array (Illumina) at AKESOgen Inc. Samples were randomized to avoid introduction of batch/chip effects. All sample preparation/processing was carried out according to the Illumina kit protocols. Epigenetic age (eAge) was estimated by applying Universal chronological clock and SVM-based multi-species multi-tissue mortality clock models to methylation β values. CpG sites with a detection p-value > 0.01 in at least 25% of samples were excluded. For the remaining CpGs, the mean methylation level and Shannon entropy were calculated individually for each sample. To compare eAge, mean methylation, and Shannon entropy among different WAT types (donor WAT Before transplantation, donor WAT After transplantation, and Native WAT), we used an ANOVA model with WAT type and donor ID as covariates within each experimental group (young-to-young, young-to-old, old-to-young, and old-to-old). Model coefficients and p-values for the WAT type factor were extracted, and p-values were adjusted for multiple comparisons using the Benjamini–Hochberg (BH) method. To evaluate differences in the dynamics of donor WAT eAge during transplantation across experimental groups, we applied an ANOVA model for each pair of experimental groups, including transplantation stage (“Before” and “After”), experimental group, and their interaction as covariates. The coefficient of the interaction term and its corresponding p-value were extracted, and p-values were adjusted for multiple testing using the BH method.

### RNA sequencing

RNA sequencing was performed at Novogene. Total RNA isolated as described above was checked for quality using an Agilent 2100 Bioanalyzer. Samples that passed QC were paired-end sequenced on an Illumina NovaSeq 6000 with 150 bp read length. Reads were mapped to a mouse genome (GRCm39) with STAR (version 2.7.11b) and counted via featureCounts (version 2.0.6). To filter out non-expressed genes, we required at least 10 reads in at least 20% of samples. Differentially expressed genes between donor’s WAT before and after transplantation were identified separately for each experimental group using ANOVA model with donor’s ID included as a covariate through edgeR package [94]. Genes with differential change of expression after transplantation in donor’s WAT across experimental groups were identified for each pair of experimental groups using ANOVA model with stage of transplantation (“before” and “after”), experimental group, and their interaction included as covariates. Model coefficient of interaction term together with the corresponding p-value was used to characterize genes with distinct effect induced by transplantation across groups. P-values were adjusted for multiple testing with the BH method.

### Transcriptomic signature analysis

We conducted functional enrichment analysis to characterize the transcriptome changes in donor’s WAT induced by transplantation across different experimental groups, and compare them to established molecular signatures of aging, mortality, and lifespan regulation. We applied reference signatures from tissue-specific aging biomarkers of liver, kidney, and brain, and from multi-tissue biomarkers of chronological age, relative (lifespan-adjusted) age and expected mortality, unadjusted and adjusted for chronological age [17, 28]. Additionally, we included hepatic signatures of expected maximum and median lifespan in rodents as well as signatures of individual longevity interventions, such as caloric restriction, genetic models of growth hormone deficiency, and rapamycin (https://pubmed.ncbi.nlm.nih.gov/31353263/). For each experimental transplantation group, we ranked gene expression changes during transplantation using a signed log-transformed p-value metric estimated through differential expression analysis:

−log(pv)×sgn(lfc),

where pv and lfc are p-value and logFC of a certain gene, respectively, and sgn is the signum function (equal to 1, −1 and 0 if value is positive, negative or equal to 0, respectively). Ranked gene lists were then subjected to gene set enrichment analysis (GSEA) using the fgsea package in R, with 10,000 permutations and multilevel Monte Carlo sampling. Gene sets were drawn from the HALLMARK, KEGG, and REACTOME collections of the Molecular Signatures Database (MSigDB). The same enrichment pipeline was applied to reference signatures of aging, mortality, and longevity interventions. Individual p-values were adjusted for multiple testing with the BH method. We computed Spearman correlations between normalized enrichment scores (NES) to quantify similarities between signatures of transplantation and reference gene expression biomarkers.

### Transcriptomic age analysis

Filtered RNA-seq data were processed using Relative Log Expression (RLE) normalization, log transformation, and scaling. Missing expression values for clock genes absent from the dataset were imputed using their precomputed average values. Normalized gene expression profiles were then centered to the median profile of young samples derived from donor WAT before transplantation.

Transcriptomic age (tAge) for each sample was estimated using Bayesian Ridge-based multi-tissue transcriptomic clocks of chronological age and expected mortality [17]. Pairwise differences in tAge across WAT groups were evaluated separately for each experimental group using a mixed-effects model, with both the mean and standard deviation of tAge predictions included, and donor ID and batch included as technical covariates. To assess differences in donor WAT tAge dynamics following transplantation across experimental groups, tAges were first adjusted for chronological age. Specifically, a model mapping chronological age to tAge was fitted using sham control samples and then applied to other samples to generate expected tAge values. The difference between observed and expected tAge was calculated for each sample, and for each pair of experimental groups, a mixed-effects model was fitted with transplantation stage (Before vs. After), experimental group, and their interaction as fixed effects, and batch as a technical covariate. P-values for the interaction terms were adjusted for multiple testing using the BH method, and these adjusted values were used to determine statistical significance of differences in tAge dynamics across groups.

To estimate the contribution of each gene to tAge changes during transplantation, we multiplied its log fold change (logFC) by the corresponding coefficient from Elastic Net-based transcriptomic clock, calculated separately for each experimental group. The statistical significance of each gene’s contribution was derived from the corresponding differential expression analysis. Comparisons of gene contributions to tAge dynamics across experimental groups were performed using Pearson correlation analysis.

To identify genes whose expression changes during transplantation were significantly associated with tAge dynamics, we analyzed samples from donor WAT collected before and after transplantation. A linear model was fitted using the edgeR package [94], with tAge difference, transplantation stage, and donor ID included as covariates. Model coefficients and p-values for the tAge difference term were extracted, and p-values were adjusted for multiple testing using the BH method. Genes with an adjusted p-value < 0.05 were considered significant signatures of tAge dynamics deviation during donor WAT transplantation. Pathway enrichment analysis for these genes was performed separately for those positively and negatively associated with tAge dynamics using Fisher’s exact test in the clusterProfiler package [95], based on KEGG ontology. Resulting p-values were adjusted using the BH method, and KEGG terms with an adjusted p-value < 0.05 were considered statistically significant pathway signatures of tAge-associated genes.

Module-specific transcriptomic clocks of chronological age and expected mortality were applied to the scaled relative gene expression profiles using the same framework. The resulting tAge estimates from each module-specific clock were standardized within each experimental group. For each module-specific clock and experimental group, an ANOVA model with donor ID as a technical covariate was used to compare tAge between WAT samples collected before and after transplantation. Resulting p-values were adjusted for multiple comparisons using the BH method.

### Tissue preparation for histology and immunostaining

Adipose tissue samples fixed in 3.5% paraformaldehyde overnight were washed with PBS and transferred to 70% ethanol for storage. Samples were paraffin-embedded and subjected to histopathological staining at the Harvard Rodent Histopathology Core. Hematoxylin and eosin (H&E) and Masson’s Trichrome stainings were performed. An Automated image analysis was performed using the Segment Anything Model (SAM), in order to quantify adipocyte size by averaging 50–200 randomly selected adipocytes per sample, enabling both between-group comparisons and within-animal assessments of graft versus native adipocyte size. Collagen deposition was quantified using SAM applied to Trichrome Masson-stained slides. Images were captured at 10× magnification, with three random fields per sample analyzed.

### Adipocyte lipid droplet quantification

Adipocyte lipid droplet size was quantified from H&E-stained sections using automated segmentation with the Segment Anything Model (SAM) employing the ViT-H architecture [96]. Images acquired at 20× magnification were processed using custom Python scripts with the following parameters: minimum mask region area of 0 pixels, predicted intersection over union threshold of 0.5, and stability score threshold of 0.8. Adipocyte lipid droplets were identified as regions with mean RGB values ≥190 for all color channels and areas exceeding 10,000 pixels^2^. Masks touching image borders within 20 pixels were excluded to eliminate incomplete adipocytes. Overlapping masks were resolved by retaining the largest mask. Cell nuclei were distinguished from adipocytes based on their characteristic purple staining (RGB values: R ≤150, G ≤150, B ≤200) and smaller size (<5,000 pixels^2^). The cross-sectional area of each adipocyte was recorded in pixels^2^. Adipocyte droplet size values were log-transformed prior to statistical analysis to approximate a normal distribution. For each mouse, the mean and standard deviation of droplet size (in the log scale) were calculated, along with the corresponding standard errors. A mixed- effects model using the REML method was applied to assess statistical differences in both the mean and the standard deviation of droplet size before and after transplantation individually for each group. Pairwise differences in adipocyte size dynamics across experimental groups during transplantation were evaluated using a mixed-effects model with transplantation stage, experimental group, and their interaction as fixed effects. The p-value for the interaction term, adjusted for multiple comparisons using the BH method, was used to determine the significance of differences in changes in adipocyte size mean and standard deviation following transplantation between experimental groups.

### Collagen deposition quantification

Collagen content was quantified from Masson’s trichrome-stained sections by identifying blue-stained collagen fibers. Pixels were classified as collagen-positive when RGB values fell within the range of 170-220 for red, 190-240 for green, and 210-255 for blue channels. The total number of collagen-positive pixels was calculated for each image field. Collagen content was expressed as the proportion of collagen-positive pixels relative to the total tissue area after excluding white spaces corresponding to adipocyte lipid droplets. All image analyses were performed using Python with OpenCV and NumPy libraries, and morphometric data were exported as CSV files for subsequent statistical analysis. Collagen content values were log-transformed prior to statistical analysis to approximate a normal distribution. Changes in collagen content during transplantation, as well as differences in these dynamics across experimental groups, were assessed using the same statistical approach described previously for adipocyte droplet size.

### Mitochondria Immunostaining

For the mitochondria morphology analyses, we performed an immunofluorescence assay in paraffin slices of 100 and 15 μm, using the previously described protocol [97]. The scWAT sections were blocked in 5% Bovine Serum Albumin (BSA) with 0.5% Triton X-100 for 1 hour at room temperature before being incubated with the primary antibody. For the mitochondria staining, we used the primary antibody Tomm20 (Novus, NBP2-67501, lot number HP0128) diluted 1:100 in PBS Triton X-100 0.25%, incubated overnight at 4°C in a humidified chamber. Sections were washed in PBS 1x before being incubated with secondary antibody Alexa Fluor anti-rabbit 568 (1:500, Thermo, A21244, lot 2659317) with DAPI (1:1000, Sigma-Aldrich, D9542) diluted in 0.1% Triton X-100. Slides were mounted with ProLong Glass Antifade Mountant (Invitrogen, P36980, Lot 2801574) and kept at 4 °C overnight before imaging. All tissues from the same group (WAT Before, After and Native) were stained within the same batch to ensure maintained consistency for image acquisition and analysis.

### Imaging and mitochondria morphology quantification

To assess mitochondrial properties, we performed immunostaining of paraffin-embedded sections using an antibody against Tomm20. Initial imaging of 100 μm sections confirmed homogeneous mitochondrial labeling throughout the adipocyte cytoplasm, and after that, we used 15 μm sections for quantitative analysis (Figure 3I). To exclude infiltrating immune cells, we focused exclusively on mitochondria within the cytoplasmic compartment, omitting those near nuclei (Supplementary Figure 3B). Following segmentation of cytoplasmic mitochondria, we quantified their numbers, as well as total volume and sphericity as readouts of mitochondrial morphology. Because cell boundaries were not always clearly discernible, we estimated mitochondrial density by dividing the total number of segmented mitochondria by the total cytoplasmic volume of adipocytes in the region of interest. Three regions per tissue sample were randomly selected and imaged using a Yokogawa CSU-W1 spinning disk confocal unit (50 μm pinhole size) mounted on a fully motorized Nikon Ti2 inverted microscope equipped with a Nikon linear-encoded motorized XY stage and a Physik Instrumente piezo Z-motor integrated into the nosepiece drive. An Andor Zyla 4.2 Plus sCMOS (6.5 μm pixel size) monochrome camera was used together with a Nikon Plan Fluor 40×/1.3 NA oil-immersion objective (Cargille Type 37 immersion oil). Images were acquired at 1×1 binning, 12-bit depth (Gain 1). Excitation for confocal imaging was provided by solid-state, directly modulated diode lasers launched through a Nikon LUN-F laser combiner, using the 405 nm, 488 nm, or 640 nm laser lines depending on the fluorophore. A hard-coated Semrock Di01-T405/488/568/647 multiband dichroic mirror was used for all laser-based channels. Green fluorescence excited with the 488 nm laser was collected using a Chroma ET535/36 nm emission filter, and far-red fluorescence excited with the 640 nm laser was collected through a Chroma ET705/72 nm emission filter. No software-based autofocus routines were used. For image acquisition, Nikon Elements AR (version 5.41.02) was used. Z-stacks were collected using either the Nikon nosepiece drive or the PI piezo Z-motor. For each field of view, images were acquired sequentially by channel, with the shutter remaining open during exposure. For each sample, a complete optical volume was acquired, typically between 41 and 50 Z-slices collected at a 0.3 μm step size. Data was saved in ND2 format. Prior to morphological analysis, all images were pre-processed in Fiji. For standardization of three-dimensional dimensions across samples, each image was cropped to include 41 optical slices, corresponding to a total depth of 12.3 μm. Image segmentation and object detection, such as mitochondria, nuclei, and cell membranes, were performed using Arivis software. Cell membranes were identified by the autofluorescence signal captured with the 488 nm laser. The three-dimensional mitochondrial morphology was assessed in terms of mitochondrial volume and sphericity. Mitochondrial volume values were log-transformed before statistical analysis to approximate a normal distribution. Changes in mitochondrial volume and sphericity during transplantation were assessed using the same statistical approach described previously for adipocyte droplet size.

### Fibrosis assessment

White adipose tissue (WAT) samples from all experimental groups were submitted to the Rodent Histopathology Core at Harvard Medical School for blinded fibrosis evaluation. A trained veterinary histopathologist, blinded to donor age, recipient age, and transplantation group, performed qualitative and semi-quantitative scoring of fibrosis. All slides were evaluated under identical conditions, and fibrosis scores were recorded and returned to the research team without disclosure of sample identity until the completion of analysis.

## Supplementary Material

1

## Data Availability

All the data will be publicly available upon publication.
