# Supplementary material for: Rejuvenation of white adipose tissue in a longitudinal heterochronic transplantation model
Source: bioRxiv. 2025 Dec 29:2025.12.28.696721. Preprint. [Version 1] doi: 10.64898/2025.12.28.696721 (PMC12776278; doi:10.64898/2025.12.28.696721)
Supplement: 1 [file NIHPP2025.12.28.696721v1-supplement-1.pdf]

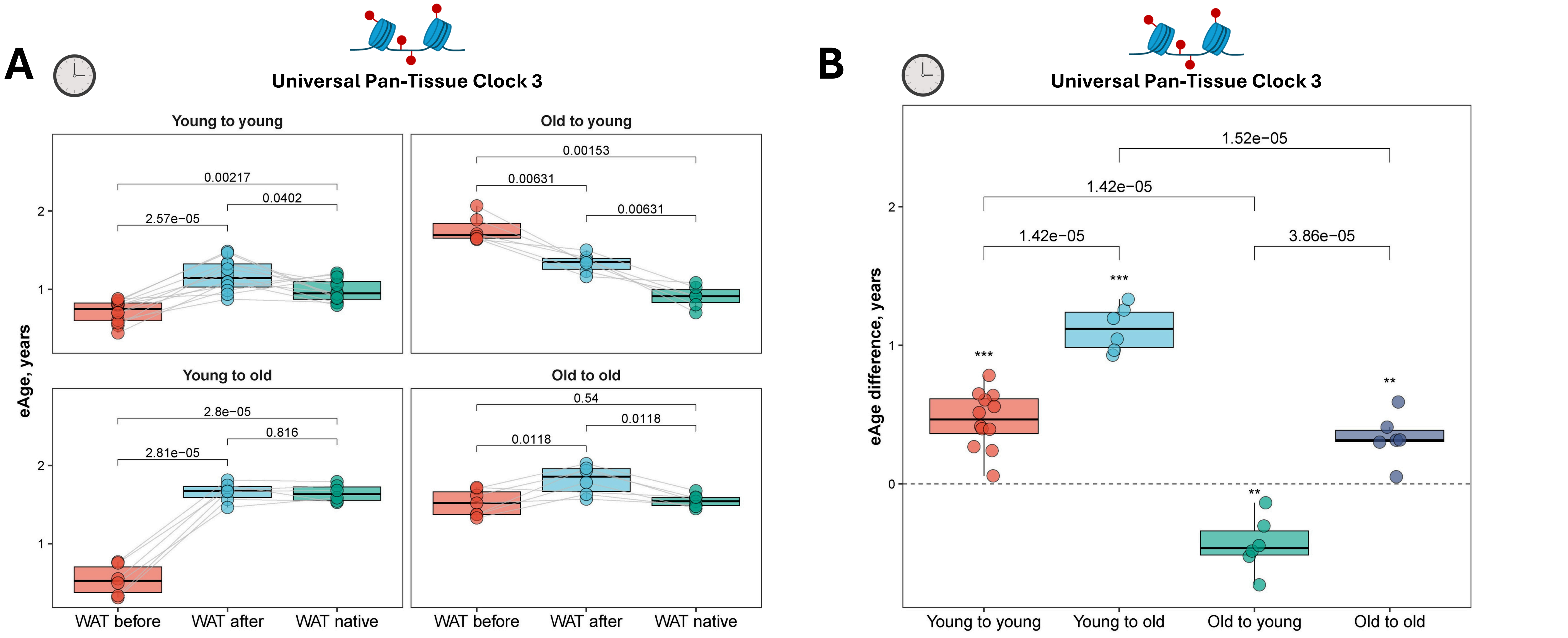

bioRxiv preprint doi: https://doi.org/10.64898/2025.12.28.696721; this version posted December 29, 2025. The copyright holder for this preprint (which was not certified by peer review) is the author/funder, who has granted bioRxiv a license to display the preprint in perpetuity. It is made available under aCC-BY 4.0 International license.

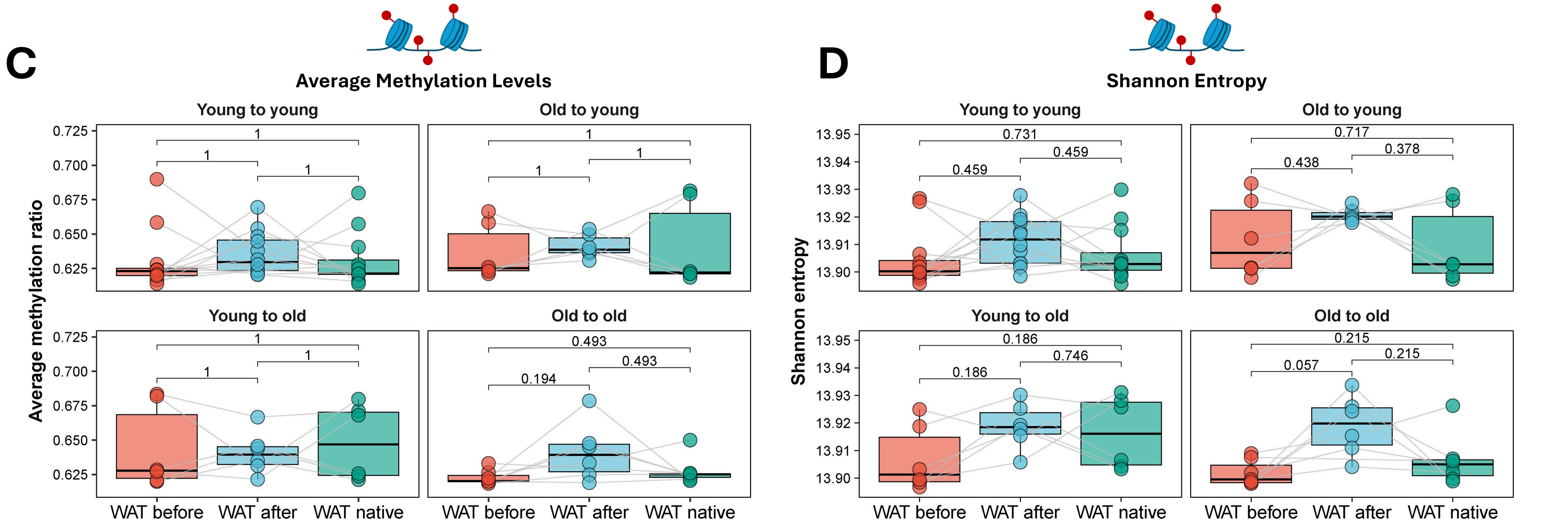

bioRxiv preprint doi: https://doi.org/10.64898/2025.12.28.696721; this version posted December 29, 2025. The copyright holder for this preprint (which was not certified by peer review) is the author/funder, who has granted bioRxiv a license to display the preprint in perpetuity. It is made available under aCC-BY 4.0 International license.

Supplementary Figure 1. Epigenetic and transcriptomic aging clocks reveal rejuvenation of old adipose tissue following heterochronic transplantation.

(A) Epigenetic age (eAge) of WAT samples before and after transplantation across all experimental groups according to the Universal Clock 3 predictions. Mouse ID was included in the statistical model as a covariate to ensure paired comparison of eAge dynamics.

(B) Change in epigenetic age (eAge) of WAT samples before and after transplantation across all experimental groups according to Universal Clock 3. Asterisks reflect statistical significance (BH-adjusted p-values) of eAge changes during transplantation within individual groups, whereas the significance of pairwise comparisons in eAge dynamics between groups is denoted in text.

(C-D) Average methylation levels **(C)** and Shannon entropy **(D)** of WAT samples before and after transplantation across all experimental groups. Mouse ID was included in the statistical model as a covariate to ensure paired comparison of average methylation levels and Shannon entropy.

Boxplots: center line indicates median; box limits, interquartile range; whiskers, ±1.5× IQR. Unless specified otherwise, statistical differences between groups are assessed with ANOVA and adjusted for multiple comparisons with the Benjamini-Hochberg approach.

*** p.adjusted< 0.001, ** p.adjusted < 0.01, * p.adjusted < 0.05.

bioRxiv preprint doi: https://doi.org/10.64898/2025.12.28.696721; this version posted December 29, 2025. The copyright holder for this preprint (which was not certified by peer review) is the author/funder, who has granted bioRxiv a license to display the preprint in perpetuity. It is made available under aCC-BY 4.0 International license.

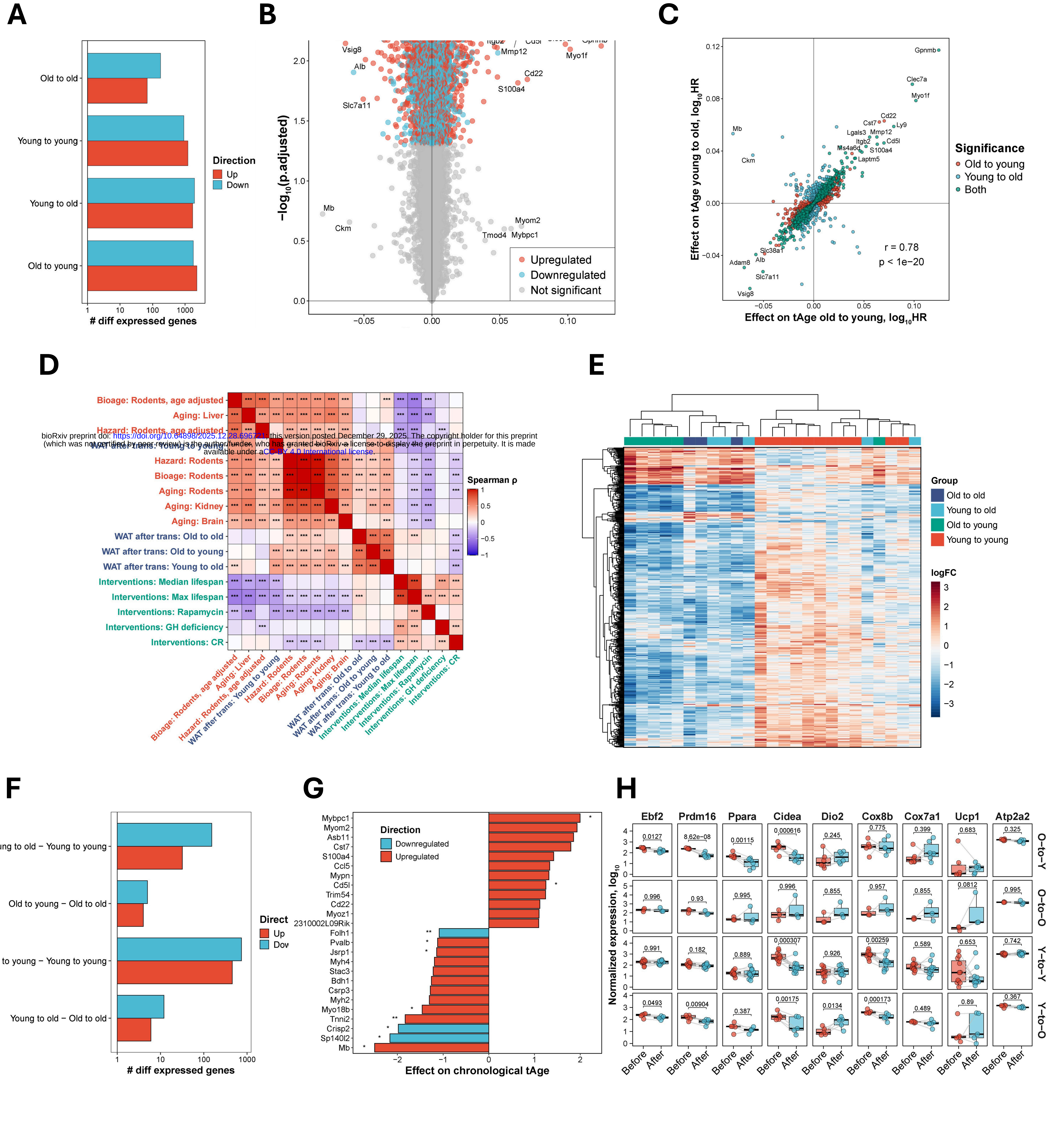

bioRxiv preprint doi: https://doi.org/10.64898/2025.12.28.696721; this version posted December 29, 2025. The copyright holder for this preprint (which was not certified by peer review) is the author/funder, who has granted bioRxiv a license to display the preprint in perpetuity. It is made available under aCC-BY 4.0 International license.

Supplementary Figure 2. Transcriptomic signatures underlying adipose tissue rejuvenation indicate activation of known and novel thermogenic pathways

(A) Number of differentially expressed genes (p.adjusted < 0.05) in a donor's WAT after transplantation relative to their respective pre-transplantation baselines for each experimental group. Barplots indicate the numbers of differentially expressed genes, and colors denote the direction of changes.

(B) Volcano plot highlighting genes altered during old-to-young transplantation and their contribution to tAge according to the EN rodent multi-tissue Transcriptomic Mortality Clock; genes contributing to decreased and increased expected hazard are shown on the left and right, respectively. The direction of expression changes after transplantation is reflected with color.

(C) Gene expression changes induced by transplantation in a donor's WAT that contribute to the change in tAge for young-to-old and old-to-young groups. R indicates the correlation coefficient, and p indicates the significance (p-value) of the observed correlation.

(D) Pathway-level correlation of WAT transplantation signatures (blue), signatures of aging, mortality (red), and lifespan-extending interventions (green). Pairwise Spearman correlations were calculated based on NES values determined for each signature via GSEA. The correlation coefficient is reflected with color, and asterisks reflect statistical significance (BH-adjusted p-values).

(E) Hierarchical clustering of transcriptional responses in WAT samples before and after transplantation across all experimental conditions. The direction of expression change is reflected with color.

(F) Number of genes with differential change of expression in a donor's WAT samples after transplantation relative to their respective pre-transplantation baselines across all experimental groups. Barplots indicate the numbers of differentially expressed genes, and colors denote the direction of changes.

(G) Top genes contributing to increased and decreased tAge during transplantation in the old-to-young vs young-to-young group, according to the rodent multi-tissue Transcriptomic Chronological Clock. Color and asterisks denote direction and statistical significance (adjusted p-value) of difference in expression dynamics between groups, respectively.

(H) Normalized expression dynamics (in log scale) of established brown adipocyte thermogenic markers in WAT samples before and after transplantation across all transplantation models.

bioRxiv preprint doi: https://doi.org/10.64898/2025.12.28.696721; this version posted December 29, 2025. The copyright holder for this preprint (which was not certified by peer review) is the author/funder, who has granted bioRxiv a license to display the preprint in perpetuity. It is made available under aCC-BY 4.0 International license.

Boxplots: center line indicates median; box limits, interquartile range; whiskers, ±1.5× IQR. Unless specified otherwise, statistical differences between groups are assessed with ANOVA and adjusted for multiple comparisons with Benjamini-Hochberg approach.

*** p.adjusted< 0.001, ** p.adjusted < 0.01, * p.adjusted < 0.05, ^ p.adjusted < 0.1.

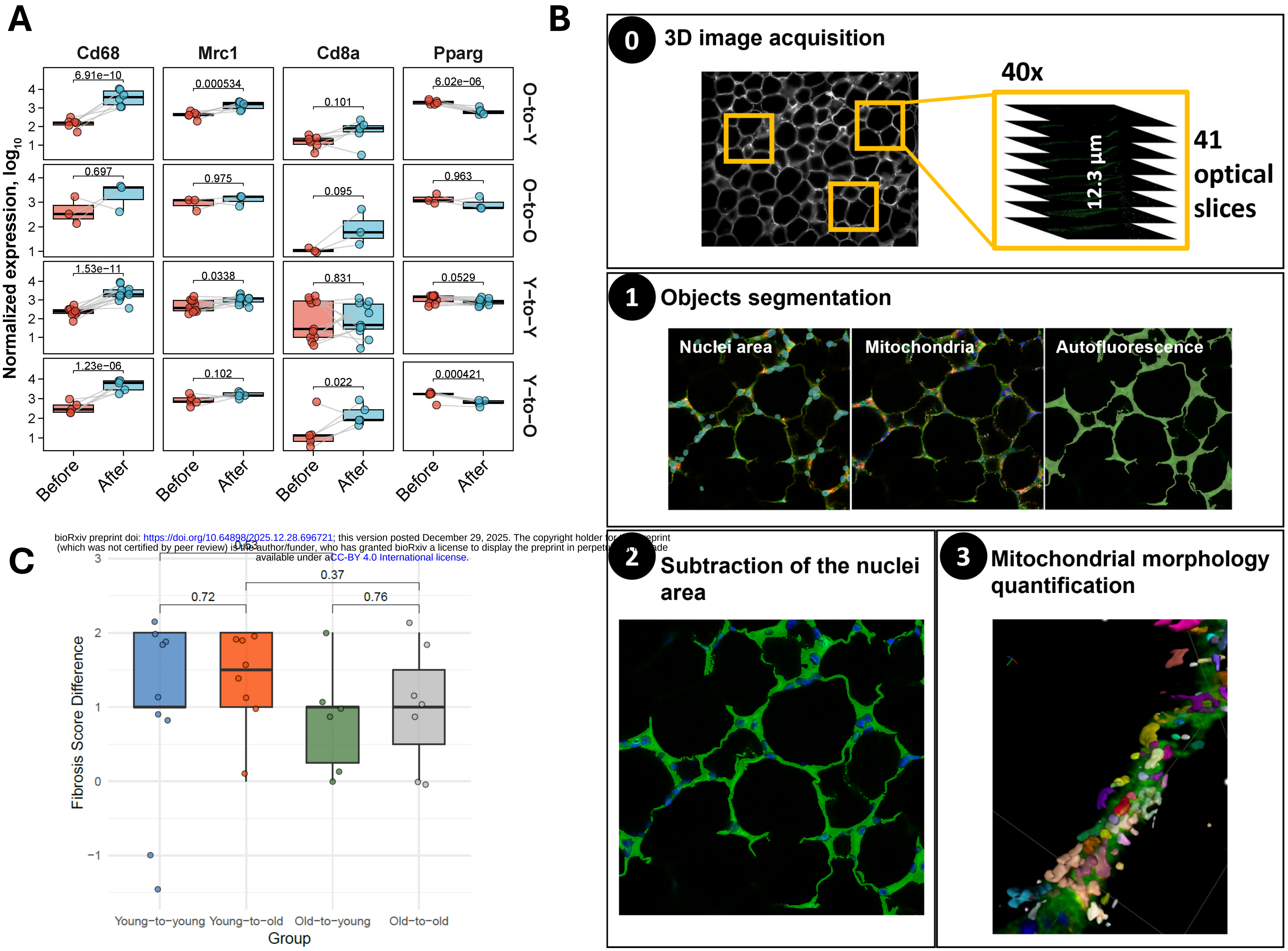

bioRxiv preprint doi: https://doi.org/10.64898/2025.12.28.696721; this version posted December 29, 2025. The copyright holder for this preprint (which was not certified by peer review) is the author/funder, who has granted bioRxiv a license to display the preprint in perpetuity. It is made available under aCC-BY 4.0 International license.

bioRxiv preprint doi: https://doi.org/10.64898/2025.12.28.696721; this version posted December 29, 2025. The copyright holder for this preprint (which was not certified by peer review) is the author/funder, who has granted bioRxiv a license to display the preprint in perpetuity. It is made available under aCC-BY 4.0 International license.

Supplementary Figure 3. Histological characterization reveals lipid droplet size as a central feature of adipose tissue rejuvenation.

(A) Normalized expression dynamics (in log scale) of established immune (Cd68, Mrc1, Cd8a) and adipocyte (Pparg) cell-type markers in WAT samples before and after transplantation across all transplantation models.

(B) Workflow for three-dimensional mitochondrial morphology analysis. (0) Three fields per tissue sample were randomly selected and imaged using spinning disk confocal microscopy, acquiring 41 optical slices per field to generate a 12.3 μm-deep 3D image stack. (1) Image segmentation was performed using Arivis software to identify nuclei (DAPI), mitochondria (Tomm20), and cytoplasm (autofluorescence signal from the 488 nm channel). (2) To exclude mitochondria from non-adipocyte cells, nuclear regions were subtracted, and only cytoplasmic regions distant from nuclei were analyzed. (3) Mitochondria were segmented in 3D and their morphological features quantified.

(C) Comparison of fibrosis score changes in WAT samples before and after transplantation across all experimental groups. Pairwise comparisons were performed using the two-sided Wilcoxon rank-sum test with the Benjamini–Hochberg correction for multiple comparisons.

Boxplots: center line indicates median; box limits, interquartile range; whiskers, ±1.5× IQR. Unless specified otherwise, statistical differences between groups are assessed with ANOVA and adjusted for multiple comparisons with the Benjamini-Hochberg approach.

*** p.adjusted< 0.001, ** p.adjusted < 0.01, * p.adjusted < 0.05, ^ p.adjusted < 0.1.
